# Exercise, Neuroprotective Exerkines, and Parkinson’s Disease: A Narrative Review

**DOI:** 10.3390/biom14101241

**Published:** 2024-09-30

**Authors:** Alexandra K. Mitchell, Rebecca R. Bliss, Frank C. Church

**Affiliations:** 1 Department of Health Sciences, Division of Physical Therapy, University of North Carolina at Chapel Hill, Chapel Hill, NC 27599, USA; akmitch414@gmail.com; 2 Physical Therapy of North Carolina, Durham, NC 27704, USA; rbliss.dpt@gmail.com; 3 Department of Pathology and Laboratory Medicine, School of Medicine, University of North Carolina at Chapel Hill, Chapel Hill, NC 27599, USA

**Keywords:** Parkinson’s disease, exercise, exerkines, neurodegenerative disease, Alzheimer’s disease, aerobic exercise, resistance training, neuroprotective theory, myokines

## Abstract

Parkinson’s disease (PD) is a prevalent neurodegenerative disease in which treatment often includes an exercise regimen. Exercise is neuroprotective in animal models of PD, and, more recently, human clinical studies have verified exercise’s disease-modifying effect. Aerobic exercise and resistance training improve many of PD’s motor and non-motor symptoms, while neuromotor therapy and stretching/flexibility exercises positively contribute to the quality of life in people with PD. Therefore, understanding the role of exercise in managing this complex disorder is crucial. Exerkines are bioactive substances that are synthesized and released during exercise and have been implicated in several positive health outcomes, including neuroprotection. Exerkines protect neuronal cells in vitro and rodent PD models in vivo. Aerobic exercise and resistance training both increase exerkine levels in the blood, suggesting a role for exerkines in the neuroprotective theory. Many exerkines demonstrate the potential for protecting the brain against pathological missteps caused by PD. Every person (people) with Parkinson’s (PwP) needs a comprehensive exercise plan tailored to their unique needs and abilities. Here, we provide an exercise template to help PwP understand the importance of exercise for treating PD, describe barriers confronting many PwP in their attempt to exercise, provide suggestions for overcoming these barriers, and explore the role of exerkines in managing PD. In conclusion, exercise and exerkines together create a powerful neuroprotective system that should contribute to slowing the chronic progression of PD.

## 1. Introduction

“Diseases fly from the presence of a person, habituated to regular physical exercise…” Susruta, a Physician in the Indus Valley civilization, 600 BCE [1,2].

### 1.1. Neurodegenerative Diseases and the Positive Impact of Exercise

Neurodegenerative diseases are characterized by progressive nervous system dysfunction leading to neuronal cell death. The most common neurodegenerative disorders include (given alphabetically) Alzheimer’s disease (AD), amyotrophic lateral sclerosis (ALS), Huntington’s disease, multiple sclerosis, Parkinson’s disease (PD), spinal muscular atrophy, and spinocerebellar ataxia [3,4,5,6,7]. Neurodegenerative diseases ultimately lead to movement (motor) difficulties, decline in cognitive functioning (dementia), or a combination of both. Most neurodegenerative diseases share similar pathophysiological alterations in brain tissue: the accumulation of abnormal protein aggregates in neurons, leading to neuroinflammation and progressive neuronal cell death [4,8,9,10,11,12].

Interestingly, exercise improves the symptoms of many neurodegenerative disorders, especially PD and AD [13,14,15,16,17,18,19,20,21,22,23]. Moderate to vigorous exercise can benefit brain health and cognitive function in people with neurodegenerative diseases [24,25,26,27], in stark contrast to the adverse effects associated with physical inactivity [28,29]. In human studies, sustained exercise enhances learning, memory, and executive function and reverses age-related mental deterioration [24]. Many studies report that sustained moderate exercise improves quality of life (QoL) outcomes in people with neurodegenerative diseases [24,25,26].

### 1.2. Parkinson’s Disease (PD)

**Figure 1:**
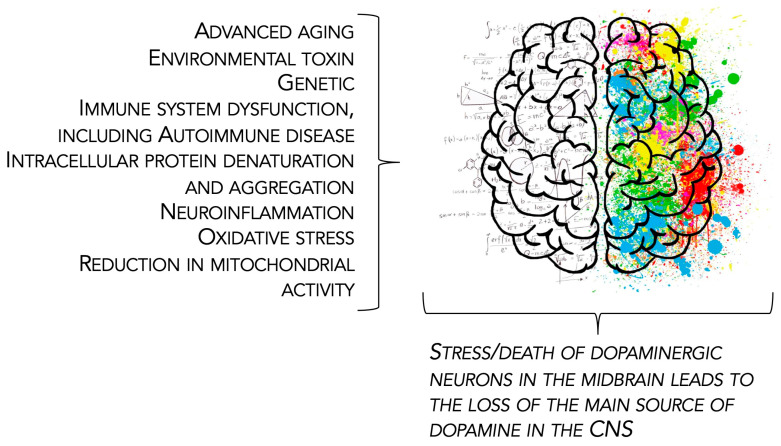
Etiology of Parkinson’s disease. The major causes of the disorder are given in alphabetical order. Abbreviation: CNS, central nervous system.

Parkinson’s disease is a neurodegenerative disorder that begins following the death of dopaminergic neurons located in the midbrain substantia nigra pars compacta [30]. The resultant degeneration of dopaminergic pathways in the basal ganglia [31,32,33,34] results in the cardinal motor signs of PD, including bradykinesia (slowness of movement), tremor (trembling in hands, arms, legs, jaw, and face), muscle rigidity (stiffness of the limbs and trunk), and impaired gait and posture [35,36,37]. Numerous non-motor symptoms of PD may include depression, psychosis, REM sleep disruption and hallucinations, difficulty swallowing and speaking, urinary problems, or constipation [38,39,40,41,42]. Most cases of PD occur sporadically and are due to a complex combination of etiologies (Figure 1).

PD is both a chronic disease process and a progressive disorder [31,32,33,34]. PD occurs most commonly in people over 60 years old. Cases of PD in younger people (<40 years old) are usually linked to specific genetic mutations. Symptoms of PD occur gradually over several years, with the rate of progression and the extent to which symptoms manifest varying from person to person. PD limits movement and results in functional instability, affecting physical well-being and QoL.

At present, PD remains an incurable disease [31,32,33,34], and treatment goals revolve around symptomatic management [43,44]. The traditional first-line therapy for PD is carbidopa/levodopa or a dopamine agonist. However, the numerous pathological events that contribute to the development of PD suggest that a multi-pronged therapeutic approach is necessary to slow disease progression.

### 1.3. Exercise and Exerkines

One of the more important treatment strategies for PD is exercise [45,46]. Regular exercise is known to benefit the health of older adults through various physiological processes. These include improving insulin sensitivity, reducing body fat, increasing cardiovascular function, enhancing cerebral blood flow and oxygenation, and improving muscle mass and strength [47]. Physical exercise promotes the synthesis/release of signaling molecules termed exerkines [48,49,50,51,52,53,54], which exert their action through autocrine, paracrine, and endocrine processes. The composition of exerkines ranges broadly from simple organic acids (e.g., lactate) [52,55] and proteolytic enzymes (e.g., cathepsin B) [23] to more complex proteins like interleukin-6 (IL-6) [56] and micro-RNAs (e.g., miR-1192) [57].

**Figure 2:**
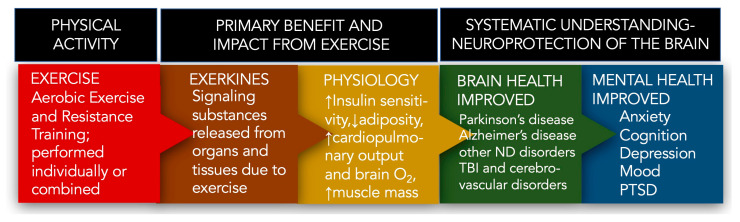
Overview of exercise and the role of neuroprotective exerkines. The molecular mechanisms underlying the protective/regenerative effects of exerkines are under active investigation. Abbreviations: ND, neurodegenerative; TBI, traumatic brain injury; PTSD, post-traumatic stress disorder.

Following acute/chronic exercise, exerkines are released from numerous tissues and organs, including skeletal muscle (myokines), the heart (cardiokines), liver (hepatokines), white adipose tissue (adipokines), brown adipose tissue (baptokines), and neurons/brain (neurokines). Exerkines migrate to their target organs during and after exercise, acting on various tissues [48,49,50,51,52,53,54]. This systemic release of exerkines facilitates communication between the brain and other organs, especially the skeletal muscles that produce myokines [19,58,59,60]. Through this communication, exercise and exerkines work together to protect the brain, providing a natural strategy to prevent brain diseases and to improve mental health (Figure 2).

### 1.4. Parkinson’s Disease, Exercise, and Exerkines

Exercise may alleviate both the physical and neurological symptoms of PD. We completed a review of the literature to determine the relationship between exercise, exerkines, and the potential to slow the progression of PD. First, this narrative review focuses on the various forms of exercise in therapy for PD. Next, we describe several neuroprotective exerkines and the neuroprotective theory. Finally, using the neuroprotective theory, we summarize how exerkines, through interorgan crosstalk, generate a protective and restorative network in the brain, verifying the potential for exercise to serve as a healthy option for managing PD.

## 2. Exercise as Therapy for Parkinson’s Disease

“Movement is a medicine for creating change in a person’s physical, emotional, and mental states.” Carol Welch-Baril, Neuromuscular Therapist

This section will focus primarily on aerobic and resistance training exercises. However, it will also include an overview of neuromotor exercise programs and flexibility exercises recognized as essential to treating PD. Numerous barriers prevent some PwP from exercising. Thus, it is necessary to describe valuable ways to overcome these obstacles. This also includes sections summarizing previous systematic and meta-reviews on the use of exercise in treating PD, and overviews of its effects on the central nervous system and motor units.

### 2.1. Defining Physical Activity and Exercise

The U.S. Centers for Disease Control and Prevention [61] defines physical activity as “any bodily movement produced by the contraction of skeletal muscle that increases energy expenditure above a basal level”. It further defines exercise as a subcategory of physical activity to sustain or improve health and fitness [61]. Data from both animal [62,63,64,65,66] and human [45,67,68,69] models suggest that engaging in moderate-to-vigorous-intensity exercise at recommended levels shows promise in slowing the neurodegeneration associated with PD. As such, exercise has increasingly become a crucial component of a holistic approach to treatment in this population.

#### 2.1.1. Exercise Intensity Defined

We define moderate-intensity exercise as physical activity that raises the heart rate to 50–70% above its resting rate. This level of exercise should feel somewhat challenging; breathing quickens but does not become labored. After approximately ten minutes of such activity, a light sweat develops, though conversation remains possible (from the Mayo Clinic [70,71] and the American Heart Association [72]). In contrast, vigorous-intensity exercise is distinctly challenging [70,71]. Breathing becomes deep and rapid, and sweating begins after only a few minutes of activity. It is typically challenging to speak more than a few words without pausing for breath.

#### 2.1.2. Calculating Maximal Heart Rate during Exercise

A widely accepted format for assessing exercise intensity involves estimating the heart rate during physical activity. To calculate the maximal heart rate, one should multiply their age by 0.7 and subtract the result from 208 [70,71,72]. For instance, for a 70-year-old individual, multiplying 70 by 0.7 results in 49, which when subtracted from 208 gives 159, representing the maximal heart rate of 159 beats per minute (bpm). Using the recommendations of the American Heart Association [72], moderate-intensity exercise should correspond to 50% to approximately 70% of the maximum heart rate (% HR_max_). For a 70-year-old, this translates to a range of 80 to 111 bpm, while vigorous-intensity exercise should range from 70% to about 85% of the maximum heart rate, equating to 111 to 135 bpm for someone aged 70 [70,71,72].

### 2.2. Aerobic Exercise

Aerobic exercise encompasses activities that increase heart rate and oxygen consumption over sustained periods, promoting cardiovascular health and overall well-being. In the context of PD, it is a cornerstone of physical activity interventions in PD management [73,74]. It has garnered considerable attention for its potential to alleviate motor symptoms and enhance cognitive function. Alberts and Rosenfeldt [75] emphasize that aerobic exercise is a universal prescription for PD management, offering benefits such as improvements in motor symptoms, balance, and overall QoL.

#### 2.2.1. Motor Symptoms

Aerobic exercise enhances cardiovascular and respiratory function, leading to better overall fitness, improved functional capacity, and increased endurance for daily activities. The American Physical Therapy Association (APTA), in their clinical practice guideline on Parkinson’s disease, encourages physical therapists to implement moderate-to-vigorous-intensity aerobic exercise (approximately 60–85% HR_max_) to improve oxygen consumption (VO_2_), reduce motor disease severity, and improve functional outcomes [76]. Several studies revealed reduced motor decline as measured by the Unified Parkinson’s Disease Rating Scale (UPDRS) [45,67,69,77]. Aerobic exercise as an intervention has also been shown to improve scores across various domains (gait, balance, and ADLS) [78]. It is often measured with gait-related outcomes, including the 6-Minute Walk Test [68].

Interestingly, and helpfully, similar results are seen across studies regardless of which mode of moderate-to-vigorous-intensity aerobic exercise is used, including treadmill walking [67], stationary cycling [45,79], a brisk walking and balance program [69], aquatic therapy [77,80], Nordic walking [81], and even relatively new methods such as agility exergaming [68]. It is reasonable to hypothesize that other forms of exercise performed at similar intensity levels, such as outdoor cycling, rowing, stair climbing, dance and others, might have similar effects [82].

#### 2.2.2. Brain Structure and Cognitive Symptoms

Aerobic exercise has been shown to improve mood and cognition in otherwise healthy older adults [83,84]. A few recent studies have extended these findings, demonstrating similar benefits in individuals with PD [85,86,87,88]. Evidence also points to aerobic exercise having beneficial effects on brain structure and cognitive function over time.

Van der Kolk et al. [45] first described the Park-in-Shape study, and they reported a large improvement in off-state MDS UPDRS scores on the stationary home-trainer bicycle (30–45 min beginning with 50% to 70% HR_max_ and gradually increasing to 80% HR_max_) compared to the control group. Johansson et al. [46], in a further assessment of the Park-in-Shape trial, concluded that aerobic exercise was associated with heightened functional connectivity between the anterior putamen and the sensorimotor cortex in its experimental groups. Compared to stretching controls, aerobic exercise mitigated global brain atrophy and contributed to enhanced cognitive control [46]. Another study suggested that aerobic exercise can change the mesolimbic dopaminergic pathway and increase evoked dopamine release in the caudate nucleus [89]. This indicated that the therapeutic benefits of exercise are perhaps related to corticostriatal plasticity and enhanced dopamine release [89]. Separately, in the Study in Parkinson Disease of Exercise (SPARX), Schenkman et al. [67] compared vigorous (80–85% Vmax) and moderate (60–65% Vmax)-intensity treadmill exercise groups to a control group. The vigorous-intensity group was found to reach targeted improvement in UPDRS scores [67].

Niemann et al. [90] found that cardiovascular interventions and motor exercises performed over six months, compared to stretching and relaxation controls, positively impacted the volume of basal ganglia nuclei and, by extension, appeared to mitigate age-related decline in cognitive functions in PwP. A more recent study by Marusiak et al. [91] revealed that eight weeks of aerobic interval training on a cycle ergometer (performed at 60–75% HR_max_) demonstrated not only an improvement in executive function for their PD intervention group but also in psychomotor domains such as mood and upper-extremity bradykinesia. Furthermore, Valenzuela et al. [92] showed that walking, when combined with cognitive tasks (known as dual-tasking) in PwP, not only demonstrated improved velocity and stride length during gait but also experienced a clinically significant improvement in perceived QoL.

A proof-of-concept study by de Laat et al. [93] evaluated the dopaminergic system of thirteen patients with mild, early PD before and after a 6-month vigorous-intensity exercise program [93]. In these individuals, exercise at an average of 80% of calculated HR_max_ led to a notable increase in dopamine transporter availability in both the substantia nigra and putamen—this was contrary to the expected decrease. Additionally, exercise caused a significant rise in neuromelanin concentration in the substantia nigra, reversing the anticipated decline [93]. Both results suggest enhanced functionality of the remaining dopaminergic neurons post-exercise and, by extension, indicate that vigorous-intensity exercise has neuromodulator and neuroprotective effects in these PwP.

### 2.3. Resistance Training

Resistance training constitutes a fundamental component of exercise interventions for PwP, aiming to improve muscle strength, endurance, and functional capacity [94]. By targeting specific muscle groups affected by PD-related rigidity and weakness, resistance training contributes to mitigating motor symptoms and enhancing overall physical performance [95]. Traditional resistance training modalities, such as weightlifting, resistance band exercises, and machine-based workouts, can be adapted to accommodate the needs and abilities of individuals with PD [96]. These exercises typically involve multiple sets of controlled repetitions targeting major muscle groups, including the upper and lower extremities, core, and back. Resistance training programs are tailored to individual baseline fitness levels and progressively adjusted to challenge muscular strength and endurance over time [97].

#### 2.3.1. Motor Symptoms, Disease Severity, and Motor Function

Corcos et al. [98] recommended progressive resistance training as part of a comprehensive exercise program for managing PD symptoms. According to the APTA, the rationale for including resistance training in a plan of care for Parkinson’s patients is to improve strength and power output, improve functional outcomes, and reduce motor disease severity as measured by the UPDRS when included in a multimodal exercise program [76].

Progressive resistance training programs offer significant benefits for PwP, improving strength and power whether implemented alone or as part of a multimodal program. Several high-quality studies [99,100,101,102] and systematic reviews with meta-analyses [94,103] consistently show that resistance training outperformed control groups (including pharmacologic treatment, education-based interventions, or low-intensity home exercises) in enhancing strength and power. Furthermore, resistance training with instability (RTI) showed greater benefits over traditional resistance training for improving strength and power of the plantarflexion and knee extension musculature, which are crucial muscle groups for maintaining functional gait patterns [104]. The RTI approach involved exercises like leg and chest presses performed on unstable devices, leading to superior results as measured by electromyography signals during isometric contractions. Silva-Batista et al. [104] found significant improvement in motor symptoms as measured by the UPDRS.

Multimodal interventions that include resistance training are generally more effective than non-exercise, education-based controls for improving strength and power in PD patients. However, these interventions are not always superior to other exercise programs or usual care in all studies. For example, a randomized controlled trial by Canning et al. [105] found that their multimodal exercise program, which targeted leg strength, balance, and freezing of gait, did not significantly reduce fall risk in their Parkinson’s patients. However, it did improve physical and psychological health.

Despite these mixed results in some comparisons, progressive resistance training has been demonstrated to address and improve functional capacity and mobility, as measured by various well-established outcome measures. Compared to pharmacological treatment, progressive resistance training was the favored intervention to improve Timed-Up-And-Go and 2-min sit-to-stand scores [45,89,103], as well as gait speed on the 10 m Walk Test [106] and 6-min walk test [107]. Specifically, resistance training with instability (RTI) had greater benefits for postural stability than traditional resistance training when measured by a digital balance system [104]. RTI also has been shown to restore gait automaticity during dual-task performance [108].

#### 2.3.2. Non-Motor Symptoms and Quality of Life

In addition to the more tangible physical benefits of resistance exercise for PwP are potential non-motor improvements in mental health and overall QoL. Non-motor symptoms can be very disabling in patients with Parkinson’s, given that the majority do not respond efficiently to dopaminergic treatment [109]. Resistance training, when compared with non-exercise or standard pharmacological treatment control groups, led to improvements in patient-perceived QoL [81,110,111], as well as in measures of depression [99] and anxiety [104,112] in PwP. Targeted strength training for 16 weeks improved the inspiratory and expiratory muscle strength of elderly PD patients, subsequently positively impacting breathing quality and overall QoL [111]. Additionally, as part of the Progressive Resistance Exercise Training in Parkinson’s Disease, or PRET-PD trial, researchers found significant differences in the Digit Span, Stroop Test, and Brief Test of Attention after 24 months of progressive resistance training, indicating that it may improve attention and working memory in patients with mild-to-moderate Parkinson’s [86].

Though most studies examining the effects of resistance training for PwP evaluate cognitive outcomes secondarily to motor outcomes, the effects appear to be beneficial overall. In the APTA’s Clinical Practice Guideline on Parkinson’s [76], much evidence suggests that no one mode of resistance training is superior to another in improving non-motor systems.

### 2.4. Neuromotor Exercises to Improve Gait, Posture, Balance, and Reduce Risk of Falls

Muscle rigidity, balance, and gait disturbances are hallmark features of PD, contributing to high fall risk and other functional limitations [113,114]. Furthermore, PwP have movements that are slow (bradykinesia), hesitant (akinesia), and with reduced amplitude (hypokinesia) [115,116]. Balance and gait training with neuromotor exercises are crucial components of physical therapy for individuals with PD, aiming to improve postural control, walking ability, and fall prevention. Attempting to normalize movements is an essential goal of neuromotor exercises and contributes to QoL issues with PwP.

Dedicated, individualized balance interventions should be considered essential exercises for PwP [76]. These exercises are important in PD, especially since the motor defects associated with PD cause stiffness, impaired balance, and slow movement. However, under the guidance of their healthcare team, PwP can develop a regular exercise routine that incorporates stretching, movement, and balance, collectively termed neuromotor exercises. There are numerous exercise modalities that PwP use to improve their motor deficits, including PWR!Moves [117,118], Rock Steady Boxing [119], Nordic walking [120], Dance for PD [121], tai chi [122], golf [123,124], and yoga [125]. This variety of options offers hope for significant improvement in the QoL of PwP. Thus, this form of therapy should be utilized throughout all stages of the disorder.

Frequently, patients with PD have problems such as freezing of gait [126], limb dystonia [127], continued functional motor limitations (e.g., bradykinesia and hypokinesia), and difficulty with swallowing/speech [128]. In each case, a Movement Disorder Specialist would arrange for treatment with a qualified Physical Therapist (PT), Occupational Therapist (OT), or Speech–Language Pathologist (SLP) to develop a treatment plan for each issue. We briefly highlight three such programs. LSVT (Lee Silverman Voice Treatment)-LOUD, a very useful program, enhances the voice, increases vocal loudness (by improving articulation, vocal quality, and intonation), and positively alters PwP functional communication skills [129,130]. LSVT-BIG helps to overcome bradykinesia and hypokinesia in PwP by focusing on large amplitude movements, which give bigger, faster, and increased movement precision [131]. PWR!Moves, another highly effective program, also utilizes high-amplitude functional exercises to target deficits that affect PwP [117,118]. PWR!Moves is used by certified physical therapists and community exercise instructors specializing in treating PD and can be utilized throughout all stages of disease progression [132].

Community programs such as Dance for PD [121] and Rock Steady Boxing [119] offer additional resources for PwP to engage in group exercise. Rock Steady Boxing utilizes large amplitude movement in the form of non-contact boxing. Participants in weekly boxing classes during a 12-week observational demonstrated improved QoL, balance and gait [133]. Dance offers opportunities for social connection, use of rhythm and music, multidirectional movement, novel movement, and cognitive engagement, including observation, imagery, and sequence recall [134]. Dance has been shown to improve QoL and cognitive symptoms for PwP [135,136], as well as motor symptoms [136,137,138,139]. Several studies examining the impact of Dance for PD on UPDRS demonstrated improved participant scores [137,138,139,140]. Examples of Dance for PwP include options such as Tango lessons or Dance for Parkinson’s classes.

### 2.5. Stretching and Flexibility Exercises to Reduce Muscle Rigidity

Muscle stiffness (rigidity) is a prevalent motor dysfunction in PD [141,142,143]. Muscle rigidity can occur in the major muscles of the arms, legs, and trunk/neck and may affect one side of the body more frequently in the disorder’s early stages [141,142,143]. Muscle rigidity may lead to balance issues, and constant rigidity may contribute to fatigue. Over time, muscle rigidity in the back/neck may lead to a stooped posture, increasing the risk of falling [144]. Rigidity is one of the motor aspects of PD that the neurologist will assess and track over time. If muscle rigidity is prevalent, the PwP may be less inclined to perform the all-important aerobic and resistance training exercises, favoring a more sedentary lifestyle. Furthermore, PwP must understand the critical negative impact of muscle rigidity and the potential benefits of managing rigidity through stretching and flexibility exercises, which can, at minimum, improve the QoL.

Developing a personal routine for stretching to increase flexibility is typically performed with a PT, ideally with knowledge or work experience with PD issues. Moreover, physical activity in general (e.g., unloading the groceries from the car, watering the plants outside the home, straightening up a room) is movement, and any movement could help reduce muscle rigidity. Many clinical trials use stretching and flexibility exercises as their control group [145,146,147]. Alternatively, stretching exercises are included with other exercises to evaluate the impact of exercise on PD [148]. Overall, stretching and flexibility exercises should be considered part of the daily life of a PwP.

### 2.6. Review of Systematic and Meta-Analysis Studies of Exercise for Treating Parkinson’s Disease

Flach et al. [149] reviewed UPDRS ratings from aerobic exercise for individuals with PD. From the final list of 245 full-text articles, 53 were ultimately reviewed for this study. They found a statistically significant positive change in UPDRS scores because of aerobic exercise.

Steiger and Homann [150] reviewed 28 studies using different types of exercise (including aerobic exercise, resistance training, balance, and tai chi). PD severity was reported using the Hoehn and Yahr (H&Y) scale, where 24 studies tested PD patients from stage 1 to 3, and four studies included more severely affected PD patients at stage 4. In general, 25 of 28 studies resulted in improved gait, functional mobility, and UPDRS III scores. They recommended that PD patients with H&Y stage 1–3 perform the following exercises: two to three sessions per week of 30 to 60 min at 70–80% HR_max_, two sessions of strength training with two to three sets per exercise with 10–15 repetitions, and two sessions of balance or tai chi for 15 min [150].

Choi et al. [151] performed a study using the PRISMA (Preferred Reporting Items for Systematic Reviews and Meta-Analyses) [152] and Cochrane Handbook for Systematic Reviews of Intervention [153] guidelines. The studies evaluated walking, strength, balancing, and complex exercise. They reviewed 18 exercise studies and found that PD patients who exercised showed significantly improved scores in UPDRS, UPDRS II, and UPDRS III. Overall, they found that exercise therapies improved the overall symptoms of PD, especially motor symptoms, balance, and gait [151].

Schootemeijer et al. [154] performed a scoping and systematic review of the overall health effects of exercise for PwP. They characterized 17 randomized clinical trials, concluding that the benefits of aerobic exercise for treating PD outweigh the potential risks and hazards. They found that aerobic exercise (treadmill walking or cycling on a stationary bike) at moderate-to-vigorous intensity (60–85% HR_max_) for 30 min per session and ~4 days/week, generally for H&Y stage 1–3, improved physical fitness in PwP (assessed by VO_2_ max) and had a beneficial effect on PD motor symptoms (assessed by MDS-UPDRS scores) (non-motor symptoms were not included as primary endpoints); however, the health-related QoL was not improved (assessed using the 39-item Parkinson’s Disease Questionnaire (PDQ-39)) [154].

Gamborg et al. [103] followed the PRISMA guidelines on systematic reviews of RCT studies. They identified 33 studies (18 resistance training, 14 aerobic exercise, and 1 other intensive exercise modality (OITM)) with PwP at H&Y stages ranging from 1 to 4. Resistance training involved using weight machines or free weights. Resistance training was performed at 50–80% maximum effort. Endurance training was performed using a treadmill or stationary bicycle, typically at 60–85% HR_max_. The OITM study combined aerobic, strength, balance, and range of motion with stretching exercises. The analysis of UPDRS-III scores revealed that aerobic exercise could improve motor defects in PD. The review of the present work found that resistance training and OITM exercises may improve UPDRS scores. Furthermore, the meta-analysis revealed that resistance training, not aerobic exercise, positively influenced QoL [103].

Martignon et al. [155] provided guidelines for the different stages of PD. They started with 115 publications/reports and used 50 records to generate exercise prescriptions. They used 11 studies for aerobic exercise that included participants with mild to moderate disease (H&Y 1–3). Nine studies were used to measure the effect of resistance training with participants at H&Y disease stages of 1–3. The general recommendation for exercise therapy in early-stage PD was moderate-to-vigorous aerobic exercise for 45 min per session for 3 days/week at 60–89% HR_max_ and resistance training performed 3 days/week, with an effort of 60–80% [155]. The recommendations for exercise in moderate-stage PD were aerobic exercise for 30–40 min at moderate effort (40–59% HR_max_) and resistance training at very light effort (<30%) for 2–3 days/week. In advanced-stage PD, the recommendation for aerobic exercise was daily with light effort (30–59% HR_max_) for 20 min or multiple sessions of 10 min, along with 2 days/week of resistance training, again at very light effort (<30% max). Flexibility exercises for all stages of the disease were recommended at 30 min/day [155].

Hao et al. [156] performed a systematic review of ten different exercise programs, including yoga, Taiji Qigong, treadmill training, resistance training, aquatic training, virtual reality training, musical dance training, walking training, cycling training, and Baduanjin Qigong training, on their effect on motor functions in PD. From a total of >6000 documents, they investigated 60 studies. Based on changes in UPDRS scores, they found that dance, yoga, virtual reality, and resistance training significantly improved motor function [156].

Osborne et al. [76] represent the Clinical Practice Guidelines from the American Physical Therapy Association. They reviewed 16 studies (9 high-quality and 7 moderate-quality studies) on the effect of aerobic exercise on PD. Most studies used treadmill walking or stationary cycling, and the participants were at H&Y stages 1–3. The trend suggests that a reduction in motor symptoms (using UPDRS-III scores) was found more frequently in those engaged in vigorous-intensity exercise (75–85% HR_max_) than those engaged in moderate-intensity exercise (60–75% HR_max_) [76]. Two high-quality and two moderate-quality studies showed improvements in gait-related outcomes, while other high-quality studies showed improvements in balance and activities of daily living. They reviewed 19 high-quality and 27 moderate-quality studies on the effect of resistance training on PD. UPDRS scores were improved in several resistance studies using different exercise procedures. Interestingly, six studies (five high-quality and one moderate-quality) found no difference in disease severity when comparing resistance training to the control group. Several studies endorsed the use of resistance training to improve QoL.

Li et al. [157] systematically reviewed exercise therapy in PD focused on slowing disease progression. They utilized updated PRISMA guidelines [158] and used potential indicators of PD progression, including MDS UPDRS scores, BDNF and TNFα levels, neuroimaging of dopamine receptor binding and other brain structures, and neurophysiological test outcomes. A total of 40 trials were studied and analyzed. This systematic review showed (the evidence was low to very low) that exercise may slow the progression of PD. The evidence was the effect of exercise on lower UPDRS motor scores and improved BDNF levels. Furthermore, exercise normalized brain activation, improved dopamine receptor binding, and reduced bradykinesia. By contrast, exercise did not reduce inflammatory or oxidative stress markers or improve brain volume. They provide numerous strategies for future RCTs to further strengthen and understand the mechanism behind the ability of exercise to slow the progression of PD [157].

Ernst et al. [159] performed a Cochrane Database of Systematic Reviews on the effect of physical exercise on PwP. They studied 156 randomized clinical trials (RCTs) with 7939 participants described as mainly mild-to-moderate disease stage and without cognitive impairment. They found evidence of beneficial effects of exercise on motor signs and QoL, with no apparent preference for the type of exercise. They further comment that PD-specific exercise programs may effectively treat specific motor symptoms [159].

Langeskov-Christensen et al. [160] performed a systematic review and meta-analysis of exercise and PD. They explored whether exercise had a role in preventing the disease (primary prevention), the potential to modify the disease progression as therapy (secondary prevention), and whether exercise is effective for symptomatic treatment (tertiary prevention). Regarding primary prevention, people who participate in moderate-to-vigorous exercise have a reduced risk of developing PD. Regarding secondary prevention, exercise is effective for symptomatic treatment, and when individually developed for the PwP, exercise is very cost-effective for PD therapy. Concerning tertiary prevention, they found that exercise had a disease-modifying effect on PD.

Umbrella reviews synthesize previous systematic reviews covering the same topic [153]. Several groups have performed umbrella reviews on physical exercise and PD [161,162,163,164], which validate the conclusions of the systematic studies and meta-analyses described above. As expected, the umbrella reviews focused on motor and non-motor symptoms of PD and the influence of exercise on improving QoL. The umbrella reviews confirmed that all of the exercise categories examined showed some benefit to PwP and that PwP were encouraged to use exercise to enhance QoL and to improve both motor and non-motor symptoms of PD [161,162,163,164].

The recommended duration and frequency per week of aerobic exercise (given in the next section) are consistent with several studies described above, including the largest exercise study reported [165]. Chekroud et al. [165] examined the mental health link with exercise in a cross-sectional survey of over 1.2 million adults older than 18. They found that individuals who participated in physical exercise for 45 min and three to five days/week had significantly fewer days of poor mental health per month than those not exercising [165].

### 2.7. Exercise Suggestions

The four types of exercise described here represent fundamental aspects of physical activity (exercise): cardiovascular endurance (aerobic exercise), strength (resistance training), flexibility (stretching/flexibility exercises), and balance/agility (neuromotor therapy). The safety of the patient while exercising is the first and most important concern. Before beginning an exercise program, a comprehensive evaluation by a neurologist and physical therapist, considering the stage of the participant’s PD, prior history of exercising, therapeutic drug usage, and existing medical comorbidities, is crucial in determining what exercises the PwP can safely perform.

Considering several outstanding sources [75,76,97,98,103,104,154,155,160,166,167,168,169,170], we suggest that PwP arrange their weekly exercise routine to include aerobic exercise, resistance training, neuromotor rehabilitation programs, and stretching/flexibility exercises. Martignon et al. [155] recommended exercise parameters for all stages of PD, but most studies/reviews only report parameters for early-stage to moderate-stage PD. Thus, using these resources cited above and from Section 2.6, we present a scalable range of values for aerobic exercise and resistance training for all stages of PD.

From a physical therapy perspective, the focus should incorporate a participant’s most appropriate intensity range for aerobic exercise. This plan is not a one-size-fits-all but a personalized approach considering the individual’s unique needs, ability, willingness to exercise, overall health, and stage of PD. Furthermore, a given PwP may easily reach the lower range of exertion and should be encouraged to exercise at their maximal percentage effort (% HR_max_ and % effort for aerobic exercise and resistance training, respectively). Alternatively, a PwP (regardless of the PD stage) just beginning to exercise may be best to adhere to the lower ranges recommended until their mastery of these lower levels has been achieved.

Aerobic Exercise—A moderate-to-vigorous intensity of 60–85% individualized HR_max_ for at least 120–150 min/week (40–50 min a day, 3 days a week) is recommended in early-stage PD. Early-stage PwP should start at 60% HR_max_ and, over time, aim high to reach 85% HR_max_. We encourage moderate-stage and advanced-stage PwP to strive for these moderate-to-vigorous intensity levels. However, for others, moderate intensity of 50–70% HR_max_ for 120–150 min/week (40 min a day, three days a week, or 30 min a day, five days a week) is advised for moderate-stage to advanced-stage PD [103,154,155].

Resistance Training—It is recommended to implement a progressive resistance training regimen, working up to 60–80% of maximal effort with 3 sets of 10 repetitions two days/week for early-stage PD and 50–69% of maximal effort with 1 to 3 sets of 10 repetitions two days/week for moderate-to-advanced-stage PD. These exercises are designed to improve muscle strength and function, which are often affected by PD [98,103,155,167].

Neuromotor Exercises—Patients with PD can significantly improve their quality of life (QoL) by incorporating the neuromotor exercise routines/programs they enjoy.

Stretching and Flexibility Exercises—It is important to include stretching and flexibility exercises in this routine. These exercises are designed to help reduce rigidity and are essential to a comprehensive exercise plan.

**Figure 3:**
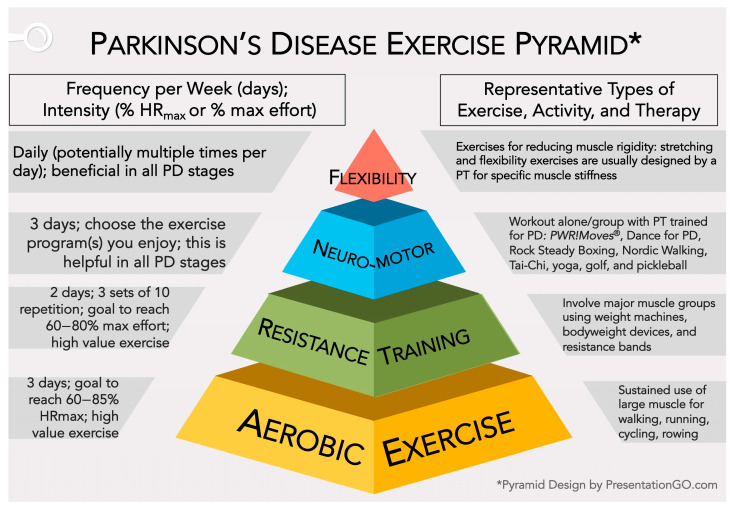
Parkinson’s disease exercise pyramid. The pyramid consists of four types of exercise: aerobic exercise, resistance training, neuromotor therapy, and stretching/flexibility exercises. On the left side are the recommended days per week, a suggested % HRmax for aerobic exercise and a % max effort for resistance training (given for a participant with early-stage PD; please see the text for suggestions at other stages of PD). On the right side is a description of the kind of exercises best represented by each category.

Figure 3 and the details above are a guide to help the PwP and Care Team (physicians and physical therapist) develop a personalized exercise plan. When followed diligently, such a plan can, over time, reduce symptoms and significantly improve the QoL for patients with PD. Please refer to these notable references for a more complete explanation of the guidelines for exercise in PD [75,76,97,98,103,104,154,155,160,166,167,168,169,170].

### 2.8. Strategies for Overcoming Barriers to Exercise for People with Parkinson’s Disease

Rafferty et al. [171] identified a cohort in the National Parkinson Foundation Quality Improvement Initiative (NPF-QII) that showed in a two-year study that early exercisers have improved outcomes compared to sedentary individuals. Therefore, it is prudent that sedentary individuals who receive a diagnosis of PD are referred to a physical therapist who specializes in PD to initiate an exercise routine specific to the individual. However, there is always time to start. Other studies have also demonstrated that when individuals with PD who have been sedentary become habitual exercisers, their QoL declines at a slower rate as compared to peer groups who remain sedentary [89]. Furthermore, with time, their brains will make adaptations that begin to look more like the brains of habitual exercisers [89].

#### 2.8.1. Reworking Aerobic Exercise

Research supports moderate-to-vigorous-intensity aerobic exercise as a strategy for improvement in motor and non-motor symptoms of PD, with the potential for vigorous-intensity exercise to have a more significant effect on slowing motor disease progression [67,76]. However, some individuals with PD may face barriers that limit their ability to participate in vigorous-intensity aerobic exercise at the recommended frequency and duration. These factors may include medical comorbidities, mobility, cognition, motivation, and environmental considerations. The potential for improvement in symptoms, overall health, and QoL with aerobic training warrants participation in whatever the individual can do safely [168,172].

Most studies examining the effects of aerobic exercise exclude individuals with advanced PD, atypical Parkinson’s disease, and Parkinsonism [171]. These populations may experience complicating factors including, but not limited to, mobility challenges requiring the use of an assistive device, cognitive challenges, or reliance on a caregiver. Although greater disease severity has been associated with increased inactivity, this is not the case for all individuals with advanced disease [173]. It is proposed that individuals with increased symptom progression may still benefit from cardiovascular exercise with modifications for safety [89].

For example, PwP with balance challenges can improve the safety of aerobic exercise by choosing exercise equipment with a seated option. This could include a recumbent bike, upper body ergometer, or even a NuStep with a chair that swivels to improve ease of transfers for individuals in a wheelchair.

Individuals with mild cognitive issues may benefit from using a group class, coach, or external cues such as a pace partner in which the participant tries to keep up with a certain pace (this feature is available on some, but not all, pieces of cardiovascular equipment). PwP with more significant cognitive impairments may require increased supervision from a caregiver to achieve moderate intensity levels.

PwP with comorbidities such as hypertension, congestive heart failure, atrial fibrillation, or renal disease (among others) may not be appropriate for high-intensity aerobic exercise. However, aerobic exercise at moderate intensity (50–70% HR_max_) or submaximal levels may be helpful for these conditions to improve function and overall conditioning [174]. A physical therapist or medical doctor may guide individuals in this category.

When determining exercise intensity, the percentage of HR_max_ may not be the most appropriate measurement for all PwP. For example, heart rate response may be blunted in individuals who are taking beta blockers. Furthermore, individuals who experience autonomic dysfunction may not demonstrate an adequate rise in heart rate with exercise [175]. For these individuals, using the Borg Rate of Perceived Exertion can serve as an appropriate alternative [176,177]. A goal of 7–8 out of 10 would correlate with vigorous activity and can be utilized as a target goal for individuals in which vigorous exercise is appropriate.

Individuals diagnosed with PD are more likely to experience a decline in physical activity related to changes in physical ability, mood, or energy levels [168]. It is not uncommon for this population to experience deconditioning; therefore, it is vital to progress gradually when initiating a cardiovascular program to improve QoL, particularly for those with a sedentary background [75,178].

#### 2.8.2. Revisiting Resistance Training

Potential barriers to resistance training include a lack of gym access, mobility challenges, and inexperience. Resistance training will benefit from modifications tailored to the individual’s needs or level of disease progression to improve participation and safety. For example, PwP with balance challenges will find seated resistance machines safer than free weights. Individuals primarily in wheelchairs may utilize ankle weights or resistance bands as part of a seated strengthening program.

PwP with inexperience will find guidance from a physical therapist or an exercise instructor with additional training in PD. Working with a personal trainer may be appropriate for individuals who may not have access to an exercise professional specializing in PD, particularly for those with earlier onset or mild presentations of PD. Individuals with moderate to advanced stages of PD will benefit from the direction of a physical therapist. For PwP who do not have access to a gym, there are various online membership programs designed for individuals with PD that one can complete from home.

As PD progresses, rigidity in the flexor groups can lead to weakness in the extensor muscle groups over time. Targeting muscles that support antigravity extension (knee extensors such as quads, hip extensors such as glutes, and trunk extension) can also improve posture.

Resistance training is not mutually exclusive to aerobic exercise. Resistance training can elicit heart rates in the target heart rate zones when performed as a high-intensity circuit. This can be achieved by choosing two or more exercises and rotating between them instead of taking a rest break between sets. By rotating exercises, one muscle group has time to rest before being rechallenged, but the body is challenged with continuous movement that may address endurance or achieve aerobic exercise components.

#### 2.8.3. Adaption of Neuromotor Exercises: Agility and Balance

Of all the domains for exercise, agility, balance, and related activities may require the greatest consideration to ensure safety and an appropriate level of challenge. Individuals who are earlier in their progression and not at risk for falls will benefit from maintaining their current ability level. These activities will ideally include multidirectional stepping, stopping, and starting and can range from tennis, pickleball, ping pong, dancing, golf, bowling, and tai chi to PWR!Moves group exercise classes. The diversity of options ensures one can find something that excites and motivates. Li et al. [179] reported that tai chi training improved motor function, gait, and balance, as measured by the UPDRS and other parameters.

Individuals at risk for falls will benefit from a physical therapist’s guidance to establish a safe balance and agility practice for the participant. For example, individuals at mild-to-moderate risk for falls may benefit from modifications to use handheld support for multidirectional stepping. This could include TRX straps, a ballet bar, a sturdy chair, or a doorway. Instead of free dance, partner dance may offer a fun and engaging alternative. Support of an exercise professional specializing in PD can also assist in maintaining safety during supervised exercise.

PwP at moderate-to-high risk for falls require the greatest attention to safety considerations. These individuals should ensure someone is present and incorporate extra precautions to perform exercises safely. For example, they may practice weight shift, marching, or multidirectional stepping with the support of their rollator while standing in front of a sturdy chair or standing at the counter with a family member close by; both scenarios consider the potential for loss of balance.

Individuals with advanced Parkinson’s who are primarily in a wheelchair or have cognitive changes will still benefit from balance training (if it can be performed safely) with a trained caregiver present. These individuals may still have to stand and turn to transfer to a commode, chair, or bed—activities that can be susceptible to falls. An example of an appropriate balance task could be standing in front of a chair with a walker while practicing weight shift with the caregiver using a gait belt.

#### 2.8.4. Refining Flexibility

Current guidelines recommend flexibility training 3×/week [180] or are unclear on the ideal dose [76]; however, optimal effectiveness may be achieved with daily training to address rigidity and improve range of motion. Given these guidelines and the need for further research, there are many choices for practical ways to incorporate flexibility training into daily practice. This could involve a daily mobility routine, activities such as yoga, or adding stretching to cool down for a different workout during the week.

Individuals with mobility challenges, particularly those who rely on an assistive device or wheelchair, may be challenged to incorporate flexibility training. However, mobility training is particularly important for these individuals to prevent contractures that can occur with prolonged sitting. Modifications to positioning can be utilized to improve accessibility. For example, stretches can be completed in a seated, supine, or prone position. An activity as simple as lying flat with arms stretched overhead or laying on one’s stomach can be an effective strategy to improve extension.

### 2.9. Examples of a Structured Exercise Strategy for Parkinson’s Disease

It is essential to consult a doctor or physical therapist before initiating a new exercise program [75], particularly for individuals new to exercise or with medical comorbidities that include, but are not limited to, cardiac disease, diabetes, or renal disease. According to the CDC, an increase in physical activity is beneficial over a sedentary lifestyle in the general population [168]. However, structured exercise beyond general activity should be encouraged for PwP due to the potential for further improvement and management of motor and non-motor symptoms. Furthermore, there is potential to modify exercise programs to tailor toward the individual’s needs, interests, and stages of disease progression. In an extensive examination of exercise testing across all stages of PD, Martignon et al. [155] recommend that PwP should engage in regular exercise and emphasize following their personalized exercise plans.

Table 1 highlights several examples of exercise programs that are unique to the individual, taking into consideration previous exercise history, current level of function, motivation, and interests. Although a future focus of this paper aims to discuss the role of exerkines specific to aerobic exercise and resistance training, Table 1 also includes examples of balance, agility, and flexibility training, widely accepted as integral components when using exercise to treat PD [180]. It is important to note that these are examples; more research is needed to determine best practice recommendations for various states of disease progression and patient-specific exercise recommendations.

### 2.10. The Effect of Exercise on the Central Nervous System (CNS) and Motor Unit in Parkinson’s Disease

Historically, exercise has improved physical fitness. Currently, exercise is used to alter the progression of PD and other neurodegenerative diseases. As research evolves, the critical relationship between exercise, CNS health, and motor unit function becomes increasingly evident.

#### 2.10.1. Exercise Enhances the Function of the CNS

Morgan et al. [181] reviewed the effect of exercise on CNS functions in murine models. Specifically, they examined the impact of exercise on circadian rhythm, central metabolism, cardiovascular function, stress responses in the brain stem and hypothalamic–pituitary axis, and movement. Of central importance to PD, exercise was found to induce adaptations in the basal ganglia. Moderate exercise showed changes in oxidative stress markers in the basal ganglia and increased production of striatal tyrosine hydroxylase, leading to the production of L-DOPA, the immediate precursor of dopamine. Moderate-to-vigorous intensity exercise also increased striatal BDNF (an exerkine to be discussed further in Section 3), a survival factor for dopaminergic neurons. Contrasting results were found in the basal ganglia as vigorous exercise disrupted vital signaling pathways in the striatum; however, it also increased striatal dopamine (D2) receptors. They conclude that exercise induces numerous changes in the brain stem, hypothalamus, and basal ganglia; further studies exploring exercise’s role throughout the lifespan would be critical [181].

Liu et al. [13] reviewed the brain’s beneficial responses to exercise. Exercise’s physiological impact can be found in the induction of neurotropic factors and neurotransmitters, the impact of myokines expressed from skeletal muscles, the reduction in neuroinflammation, enhanced mitochondrial health, and the ability of exosomes (small phospholipid vesicles) to facilitate the transport of exerkines across the BBB. They noted that exercise improved executive dysfunction, cognitive performance, and psychiatric problems; however, vigorous exercise was required to improve motor function in PD [13].

#### 2.10.2. Exercise Improves Motor Function

Perrey [182] provided an overview of how exercise positively altered motor system function. Restoring motor system function is a key goal for exercise and PD. The motor nervous system controls voluntary movement; it consists of the brain, spinal cord, and the nerves that connect these nervous system structures to the effector muscles. Moderate-intensity exercise, taking place over a sustained time period, was the most neuroprotective of motor systems [182].

Lavin et al. [183] evaluated a 16-week high-intensity resistance exercise program to gauge rehabilitation in PwP. The PD participants showed significant improvement in muscle mass, motor scores (from UPDRS), strength, power, motor unit activation, and several other indices of neurological function. They generated transcriptome-wide skeletal muscle libraries in PwP before and after the exercise program. They detected 304 genes related to remodeling and nervous system/muscle development that were upregulated and found downregulation of 402 genes that were primarily negative regulators of muscle development. These results imply that exercise promotes the remodeling of damaged motor units to improve motor function [183].

Palasz et al. [66] summarized that exercise in animal models of PD is protective and promotes the recovery of motor function. They found that exercise in animal models of PD could protect dopaminergic neurons, prevent further loss, and promote their restoration. The likely pathway of renewal was by neurotrophin activation, stabilization of intracellular calcium levels, upregulation of anti-oxidative substances, and the suppression of pro-inflammatory cytokines. Furthermore, exercise improved motor unit circuitry. Unfortunately, animal models of PD do not fully replicate PD since neurotoxins are the frequent mode for creating the clinical syndrome characterized in the mouse model of PD. Finally, progress in understanding PD can be advanced if common neuroprotective processes exist in human PD and animal models of PD [66].

Kelly et al. [184] studied the effect of resistance training on motor unit remodeling in PD. PD is an age-related neurodegenerative disease, and PwP have a more significant motor unit loss and disruption compared to age-matched peers without PD. They found that PwP had an unusual group of type I myofibrils and further reported that the advancement of these type I myofibrils led to disrupted motor unit recruitment. However, they describe that resistance training could reverse these dysfunctional motor units [184].

#### 2.10.3. Long-Term Effect of Exercise on Parkinson’s Disease

Reiner et al. [185] performed a systematic review of the long-term (>5 years) relationship between physical activity and weight gain, obesity, coronary heart disease, type 2 diabetes mellitus, Alzheimer’s disease, and dementia. Their results imply that physical activity has a positive long-term influence on all selected diseases [185].

Li et al. [186] describe the global effect of exercise on PD by examining images of the brain. They found that exercise activated the cerebellum, occipital lobe, parietal lobe, and frontal lobe, suggesting that exercise improves PD due to changes in multiple regions of the brain.

Tsukita et al. [187] studied the long-term influence of exercise in early-stage PD. They found that at baseline, regular physical activity and moderate-to-vigorous intensity exercise did not affect the clinical progression of PD. Over time, continued participation in regular physical activity and moderate-to-vigorous exercise was found to slow changes in postural and gait stability, daily living activities, and processing speed. They conclude that maintenance of regular physical activity and moderate-to-vigorous intensity exercise levels provided a better clinical course of PD [187].

### 2.11. Summary of Exercise and Introduction to Exerkines

As presented in this section, aerobic exercise and resistance training can slow the trajectory of PD progression, while neuromotor programs and flexibility/stretching exercises contribute to an improved QoL. In the next section, we describe that physical activity reduces the risk of developing PD. Part of the successful benefit of exercise in preventing PD likely arises due to the generation and release of exerkines. Thus, the remainder of the following section describes some neuroprotective exerkines.

## 3. Exercise-Induced Neuroprotective Exerkines

“Natural forces within us are the true healers of disease.” Hippocrates (460–375 BCE) [2]

### 3.1. The Association between Cardiorespiratory Fitness and the Risk of Parkinson’s Disease

Numerous studies have measured the relationship between cardiorespiratory fitness and the risk of PD. One study reported that a higher level of physical fitness reduces the risk of PD after adjustment for age and smoking [188]. Separately, the risk of PD was reduced in the patient population of most baseline recreational activities [189]. In a test population of >40,000 people, a medium level of physical activity lowers the risk of PD [190]. Furthermore, there was a decreased risk of PD in patients with heavy leisure-time physical activity compared to no physical activity [191]. By contrast, not every study found an effect of physical activity on the risk of developing PD [192]. Although there are various ways in which physical activity and cardiorespiratory fitness are determined, most studies suggest that moderate-to-vigorous-intensity physical activity reduces the risk of PD [160].

### 3.2. Neuroprotective Exerkines

Safdar et al. [26] introduced the term exerkines to represent substances released due to exercise. The ultimate goal of exerkines is to unify endocrine, autocrine, and paracrine processes, at a cross-talk between organs, tissues, and physiological systems with the CNS/brain [193]. The function of exerkines following exercise demonstrates that exercise has the potential to be disease-modifying, which can lead to a healthier brain [13,23,194]. Table 2 and the narrative below show a partial list of neuroprotective exerkines. The exerkines are presented in terms of tissue of origin, target tissue, receptor, and signaling, and they are linked to normal brain health or are related to neurodegenerative diseases/PD.

#### 3.2.1. Adiponectin

Adiponectin is mainly produced by white adipose tissue (WAT). The “gut–brain” axis interacting with adiponectin has been implicated through gut microbiota [198]. The consequences of adiponectin with its target receptors (Adipor1, AdipoR2, and T-cadherin) result in the activation of several key signal transduction pathways, most notably, AMPK, p38 MAPK, and the activation of PPARα [197]. AMPK is activated by physiological signals that indicate a negative energy balance. p38 MAPK is expressed in the brain and has functions focused on learning and memory, and it plays a vital role in synaptic regulation and function. PPARα is involved in the brain’s glutamate homeostasis and cholinergic/dopaminergic signaling. Metabolically, adiponectin is involved in glucose and fatty acid metabolism when upregulated from exercise [195]. Adiponectin has anti-inflammatory action, and there is substantial evidence that adiponectin modulates immune cell responses in both the innate and adaptive immune responses (supporting the activation of M2 anti-inflammatory macrophages) [250,251]. A dysfunction/deficiency of adiponectin with the gut–brain interface could predispose someone to a potential problem favoring the development of PD [252].

#### 3.2.2. Apelin

WAT and skeletal muscle following exercise principally synthesize apelin [48,253]. APJ is an orphan GPCR that binds the ligand apelin [199]. Apelin/APJ interaction signals an essential set of pathways, including PI3K/AKT/mTOR, ERK 1/2, and inositol, requiring kinase 1α/XBP1/C/EBP homologous protein [254]. PI3k/AKT/mTOR activation supports the survival and development of dopaminergic neurons. In contrast, the activation of ERK 1/2 induces changes in the geometry of the nucleus in response to neuronal activity in hippocampal neurons. Apelin/APJ interaction promotes several neuroprotective events, including antioxidant and antiapoptotic action, helping preserve neuronal cell loss, reduce striatum neuroinflammation, and enhance excitotoxicity [199].

#### 3.2.3. Beta-Aminoisobutyric Acid (BAIBA)

BAIBA is formed in skeletal muscle from an amino acid (valine) and a nucleic acid (thymine) [200,201,202]. This unusual metabolite is released during exercise, and BAIBA participates in glucose [255], lipid [256], and bone metabolism [257]. Interestingly, BAIBA helps down-regulate inflammation [258]. Furthermore, BAIBA helped reverse reactive oxygen species (ROS)-driven oxidative stress in mitochondria [259]. BAIBA levels were substantially increased in people performing an acute exercise routine compared to baseline [260]. The ability of BAIBA to protect mitochondria undergoing oxidative stress, combined with a potent leukocyte-derived anti-inflammatory reaction, suggests that BAIBA would offer considerable possibility to develop new/novel strategies to slow down the progression of PD.

#### 3.2.4. Beta-Hydroxybutyrate (BHB)

BHB is synthesized in the liver from fatty acids [203]. Systemically, BHB is an essential energy carrier from the liver to peripheral tissues. BHB can cross the blood–brain barrier, providing energy to neurons where glucose is reduced. BHB binds to at least two cell surface G-protein-coupled receptors, HCAR2 and free fatty acid receptor 3 [204,261]. HCAR2 is also expressed in various other cell types, including immune cells, microglia, and colonic epithelial cells, in which its activation induces anti-inflammatory effects [204]. BHB’s anti-inflammatory role was shown by inhibiting the inflammasome NLRP3 in innate immunity [262]. In the CNS, BHB is partially associated with neuroprotection mediated by the induction of BDNF in cerebral cortical neurons [263]. BHB/HCAR2 activation of Ly-6C^Lo^ monocytes and/or macrophages provides a signal to the brain for neuroprotection [204].

#### 3.2.5. Brain-Derived Neurotrophic Factor (BDNF)

BDNF is produced in the brain and skeletal muscle [48]. Moreover, from exercise, the skeletal-muscle-derived BDNF crosses the blood–brain barrier [206]. In a scenario where the degeneration of brain tissue has occurred to regrow/repair/rejuvenate neurons, BDNF fulfills a role. BDNF increases cell growth and survival, and BDNF also enhances synaptic plasticity. BDNF binds to its receptor, tropomyosin receptor kinase B (TrkB) [264]. Upon activation of TrkB, several small G proteins, including Ras, MAP kinase, PI3-kinase, and phospholipase-C-γ pathways, are upregulated, leading to neural plasticity, neurogenesis, stress resistance, and cell survival [265,266]. This suggests the comparative flexibility of TrkB in terms of pro-survival function. Not surprisingly, decreased levels of BDNF are associated with neurodegenerative diseases with neuronal loss, most notably PD, AD, multiple sclerosis, and Huntington’s disease [267]. Furthermore, reduced levels of BDNF are linked to cognitive deficits in PD [268]. By contrast, Paterno et al. [269] found that increased exercise intensity resulted in higher levels of BDNF in blood serum from PwP.

#### 3.2.6. Cathepsin B (CTSB)

CTSB is a proteolytic enzyme secreted during exercise from skeletal muscle. Although it has a catabolic function in muscle by enabling the repair of damaged muscle, CTSB can cross the blood–brain barrier [210,211]. Moon et al. showed that CTSB led to increased levels of *Bdnf* mRNA and BDNF protein expression [210]; however, CTSB did not alter hippocampal cell proliferation. Furthermore, they reported that another protein is upregulated, specifically doublecortin, which has known neuroprotective activity during neuronal migration [210].

#### 3.2.7. Fetuin-A

Fetuin-A is a hepatokine that primarily reduces inflammation in the liver to protect against liver damage [212,213,214]. Substantial research has shown that fetuin-A is a multifunctional protein with many important physiological and pathological roles. Blood-borne fetuin-A can cross the blood–brain barrier and was shown to be upregulated and neuroprotective in a mouse model of TBI [270]. In this model, fetuin-A activated Nrf2/HO-1, suppressing oxidative stress and necroptosis levels and attenuating the abnormal inflammatory response following TBI [270]. In a separate study, fetuin-A levels were reduced in human and mouse Purkinje cell PD samples [271]. Fetuin-A is a multi-tasking exercise with the primary regulator of essential cytoprotective responses in the brain, upregulating genes that can possess anti-oxidative, anti-inflammatory, and detoxifying proteins [272].

#### 3.2.8. Fibroblast Growth Factor 21 (FGF21)

FGF21 is primarily synthesized in the liver [215]. FGF21 is a hepatic signal that induces food intake and improves several metabolic parameters, such as energy expenditure and BAT thermogenesis, mainly by its action in the hypothalamus [217]. FGF21 binds to the co-receptors FGF receptor one and β-Klotho [273]. Pre-existing diabetes is a negative risk factor for developing PD, which leads to dysregulated glucose and lipid metabolism in PD. Boosting levels of FGF21 resulted in a reduction in proinflammatory cytokines and improved fuel utilization (glucose and lipid) [274]. Although FGF21 is a liver protein, it directly influences the nervous system to help regulate energy homeostasis, glucose and lipid metabolism, and insulin sensitivity.

#### 3.2.9. Glial Cell Line-Derived Neurotrophic Factor (GDNF)

GDNF is a crucial participant in developing and maintaining mid-brain dopaminergic and spinal motor neurons [218,219,220]. The Ret proto-oncogene is the signaling receptor for GDNF; however, receptor activation requires an accessory protein, GDNF family receptor α1 [218,219,220]. After engaging the receptor, GDNF promotes the survival of many types of neurons. Notably, GDNF is reduced in the substantia nigra of PD patients [275]. Short-term exercise increased GDNF levels in the spinal cord [218]. GDNF has both neuroprotective and neurorestorative potential in treating PD [275].

#### 3.2.10. Glycosylphosphatidylinositol-Specific Phospholipase D1 (GPLD1)

GPLD1 is a phospholipase that cleaves GPI-anchored cell-membrane proteins. There has been much work trying to reverse or slow the effects of aging using systematic processes like exercise [276,277]. Another approach is transferring blood plasma from young to aged animals [278]. The goal was to compare exercise and young blood plasma to improve the regenerative process and cognition in the aged brains of old animals. Horowitz et al. [221] identified GPLD1 as the critical component in the blood that transfers the effects of exercise on adult neurogenesis and cognition to sedentary aged mice. Exercise increased the levels of GPLD1 detected in mouse blood, which was similarly correlated in active older humans [221]. Horowitz et al. [221] then performed in vivo transfection of GPLD1 into mice and measured increased levels of GPLD1 in plasma, which increased neurogenesis and BDNF expression in old mice. They also demonstrate the importance of this liver-to-brain axis in improving age-related regenerative and cognitive changes [221].

One system proposed to be modified by GPLD1 was the GPI-anchored uPAR of the plasminogen-urokinase protease system [221]. A physiological regulator of the plasminogen/uPA/uPAR complex is PAI-1 [279,280]. We recently proposed a detrimental link between PAI-1 and its upregulation by neuroinflammation, which would reduce the cleavage of α-synuclein (which forms Lewy Body inclusions) by plasmin as part of the pathological process of PD [281]. Exercise reduces PAI-1 levels and, when combined with exercise, increasing GPLD1, suggesting a novel pathway to potentially reduce the impact of neuroinflammation on the progress of PD. However, further in-depth studies are needed to advance this hypothesis.

#### 3.2.11. Insulin-like Growth Factor-1 (IGF-1)

IGF-1, a neurotrophic factor, is produced in the liver, skeletal muscle, and other tissues [223,224,225]. Along with BDNF and VEGF, IGF-1 is a critical factor for brain health [224]. In this context, IGF-1 mediates the effects of exercise on brain health, protecting against brain injuries and improving memory and cognitive functions [282]. Muscle hypertrophy (i.e., resistance training) [283,284] and aerobic exercise [285] can increase levels of IGF-1 into blood following exercise. IGF-1 binds to the IGF-1 receptor on the cell surfaces of targeted tissues. Ligand binding to the α subunit of the receptor leads to a conformational change in the β subunit, activating receptor tyrosine kinase activity [286]. IGF-1 protects the nigrostriatal pathway in PD by activating essential pro-survival signaling cascades [287]. A reduction in brain insulin/IGF-1 signaling may be part of the complex pathophysiology of PD, which would support the use of anti-diabetic drugs currently being studied for their potential disease-modifying properties in PD [288].

#### 3.2.12. Interleukin-6 (IL-6)

IL-6 is one of the most well-studied inflammatory cytokines, and some IL-6 is synthesized and released from skeletal muscle during exercise [226,227,289]. Physiologically, the role of this IL-6 is to balance lipolysis. Furthermore, and of interest to the brain, IL-6 can penetrate the blood–brain barrier and behave as an anti-inflammatory agent. The binding of IL-6 to IL-6R/gp130 promotes the signaling activity of this cytokine [290]. The production of IL-10 and IL-1 receptor antagonists primarily drives the anti-inflammation action of IL-6. Furthermore, skeletal muscle IL-6 is more closely linked to metabolism than inflammation [291], partly due to the absence of NF-kB activation. It was shown that when glycogen levels are low, contracting muscle increases IL-6 levels. Thus, from exercise, IL-6 appears to be an energy sensor that is partly regulated by muscle contraction.

#### 3.2.13. Irisin (FNDC5)

Irisin is the proteolytic fragment of fibronectin type III domain-containing protein 5 (FNDC5), a type I transmembrane glycoprotein [229]. FNDC5 is produced in skeletal muscle from exercise, and irisin is released into the circulation. Physiologically, irisin (FNDC5) regulates energy metabolism by the transcriptional-peroxisome-proliferator-activated receptor gamma (PPARγ) coactivator 1α (PGC-1α)/FNDC5 pathway by activating thermogenic function in adipose tissue, mitochondrial biogenesis, and other gene-programming functions [229]. PGC-1α is a key regulator of mitochondrial quality control and energetic metabolism [292]. Interestingly, exercise induces both FNDC5 and BDNF expression in the brain, again dependent on PPARγ/PGC-1α [231]. Zhang et al. [293] studied a group of PD patients with a 12-week exercise program. The exercise PD group had elevated serum irisin levels and improved balance scores. They found that irisin protected mitochondria from oxidative stress [293]. Mechanistically, irisin activates Akt and ERK 1/2 signaling pathways, which reduces apoptosis in mitochondria with improved biogenesis [293]. These results with irisin are promising for further studies on treating PD.

#### 3.2.14. Lactate

Lactate is produced during exercise in skeletal muscle. One localized effect of lactate in newly exercised muscle is a reduction in the pH of the blood to help more rapidly re-oxygenate the muscle tissue [232,233,234]. In a different role, lactate acts as a signaling molecule in the brain by stimulating the generation of new mitochondria [294]. Lactate is a “volume transmitter” in the brain, and its receptor GPR81 (also known as HCA1 or HCAR1) promotes lipid storage in the mammalian brain [295]. Lactate is a volume transmitter in brain tissue because it distributes cellular signals supporting large neuronal ensembles [296]. It is interesting to speculate that not only is lactate fueling the brain but the lactate/GPR81 interaction may pave the path of neuroprotection by then activating downstream signaling pathways (for example, PI3k/AKT/mTOR and ERK 1/2) [294,295].

#### 3.2.15. Lac-Phe

Li et al. [235] began their search for exerkines by performing an exercise-induced metabolomics assessment of blood plasma in mice. One of the most significantly induced non-targeted metabolites was shown to be N-lactoyl-phenylalanine (Lac-Phe) [235]. They then reported that the enzyme responsible for the synthesis of Lac-Phe, CNDP2 (cytosolic non-specific dipeptidase, also known as carnosine dipeptidase 2), was found predominantly in epithelial cells and macrophages [235]. CNDP2 is found in the gut and kidney [236]. Although the mechanism is still being determined, Lac-Phe suppresses appetite and food intake [235,236]. Ultimately, the result of exercise generates Lac-Phe, which leads to a reduction in body weight. Interestingly, metformin (a drug widely used in diabetes and off-label in PD) mediates weight loss through Lac-Phe [237].

#### 3.2.16. miR451a and miR-150-5p in Small Extracellular Vesicles (sEVs)

MicroRNAs (miRs) regulate gene expression from transcript degradation and inhibit translation. Frequently, miRs are carried through the circulation using small extracellular vesicles (sEV) [239], whereby the miRs are transported as cargo [240]. Small extracellular vesicles can penetrate the blood–brain barrier. In elegant work, D’Souza tested ten males through a rigorous acute workout and measured the release into the circulation of twenty-nine well-known miRs [297]. They found that after an acute high-intensity workout, sEVs were released following exercise that contained miR-451a and miR-250-5p [297]. Interestingly, levels of miR-451a are lower in plasma in patients with depression [298]. Likewise, in a mouse study, there was an inverse relationship between low miR-250-5p and an increase in anxiety [299]. Exercise has been shown to be effective in treating depression and anxiety [300]. These results imply that miRs, bound in sEV, can be released into the circulation following exercise, and their cargo transported to the brain may be useful tools for treating depression and anxiety, which are both common non-motor events in PD.

#### 3.2.17. Monoamine Neurotransmitters (Dopamine (DA), Norepinephrine (NE), and Serotonin (5-HT))

Exercise leads to increased secretion of the monoamine neurotransmitters DA, NE, and 5-HT in the CNS [241,242,243]. Exercise increases striatal DA, which suggests that it improves executive function. It was also found to protect against neurotoxicity in an animal model of PD [63]. Thus, exercise may yield a partial neuroprotective effect on dopaminergic neurons [63,66,301]. Exercise enhances neuronal cell adaptation to stress in the neurons of the locus coeruleus, which is attributed to the up-regulation of galanin, leading to the suppression of NE [302]. The synthesis of 5-HT is affected both positively and negatively by exercise [302]. Exercise reduces the level of the 5-HT1B receptor in the dorsal raphe nuclei, which supports the hypothesis that exercise helps to relieve anxiety and helps mitigate the stress response. There is also a positive feedback loop that exercise would boost between 5-HT and BDNF, which assists in regulating synaptic plasticity and neuronal cell survival [303].

#### 3.2.18. Peroxisome Proliferator-Activated Receptor-Gamma Coactivator (PGC)-1alpha (PGC-1α)/Kynurenine (Kyn) Interaction

PGC-1α is a transcription coactivator essential in regulating cellular energy metabolism [244]. Exercise activates PGC-1α in skeletal muscle and improves mitochondrial biogenesis, fatty acid oxidation, and resistance to atrophy [292]. A part of the process is the formation of the PGC-1α-PPARα-PPARδ pathway, which leads to the activation of kynurenine aminotransferase in skeletal muscle [304]. This transferase converts Kyn into kynurenic acid [305]. Why is this important? Increased amounts of Kyn, which crosses the blood–brain barrier, are associated with depression [306]. Thus, exercise leads to PGC-1α activation in skeletal muscle along with kynurenine aminotransferase and the generation of the neuroprotective kynurenic acid (which does not cross the blood–brain barrier), which reduces stress-induced depression. As mentioned, exercise induces the up-regulation of PGC-1α and irisin (FNDC5), further highlighting the importance of PGC-1α interactions favoring a neuroprotective brain microenvironment.

#### 3.2.19. Vascular Endothelial Growth Factor (VEGF)

VEGF is a potent angiogenic and vascular permeability factor. Interestingly, VEGF also has neurotrophic, neuroprotective, and angiogenic properties through the inhibition of apoptosis and stimulation of neurogenesis [247,248,249]. VEGF also mediates multiple processes, including angiogenesis, blood–brain barrier permeability for glucose, and antioxidant activation, indirectly resulting in neuroprotection [247,248,249]. Importantly, VEGF prevents neurons from death under critical conditions such as hypoxia and glucose deprivation by binding to specific receptors, which include VEGFR-2/flk-1/KDR receptors. VEGFR-2 is the major receptor transmitting mitogenic and trophic signals in neurons [307]. Flk-1 participates in neurogenesis, hematopoiesis, vasculogenesis, and angiogenesis [308]. KDR is the main mediator of VEGF-induced endothelial proliferation, survival, migration, tubular morphogenesis, and sprouting [309]. VEGF is decreased in the substantia nigra in experimental models of PD; however, the therapeutic potential to replace the VEGF is plausible [310].

### 3.3. Exerkines and the Ability to Cross the Blood–Brain Barrier

The majority of exerkines listed in Table 2 can pass through the blood–brain barrier (BBB) into the brain, including Apelin [311], BHB [312], BDNF [313], CTSB [210,211], Fetuin-A [314], FGF21 [315], IGF-1 [316], IL-6 [317], Irisin [318], Lactate [319], mIR451a in sEV [320], mIR-150-5p in sEV [321], and VEGF [322]. PGC-1α may not be needed to cross the BBB because PGC-1α is up-regulated in neuronal inflammatory cells [244]. It was first reported that Adiponectin could not penetrate the BBB, although more recent work has shown that it does [323,324,325]. Since the brain is a target for Lac-Phe to suppress the appetite, it is logical to presume that the exerkine can navigate the BBB [235,236,237]. GDNF is already in the CNS, so it is likely not critical that it cannot pass through the BBB [326]. Dopamine cannot pass through the BBB; however, its precursor, levodopa, can enter [327]. Likewise, serotonin is not allowed free access into the brain, but its immediate precursor, 5-hydroxy-tryptophan, can pass through [328]. The BBB prevents entry of norepinephrine [328,329]. This has not been studied for BAIBA and the BBB.

### 3.4. Summary of Exerkines

Exercise-induced exerkines support several targeted pathways for the brain that allow it to renew, rejuvenate, and repair damaged/dysfunctional neuronal tissue. The following section compares aerobic (endurance-based) and resistance training (strength-based), age, sedentary lifestyle, and muscle mass on exerkine production.

## 4. Factors That Modulate the Production of Exerkines

“Look to the nervous system as the key to maximum health.” Galen (130 AD–210 AD) [2]

### 4.1. Aerobic Exercise (Endurance-Based) versus Resistance Training (Strength-Based)

Aerobic exercise (or endurance-based) and resistance training (or strength-based) activities give the two extremes of the spectrum for exercise [330]. Both exercise modalities provide substantial health benefits, but there are differences; aerobic exercise is better for reducing cardiovascular risk factors, and resistance training is better for maintaining primary metabolism and muscle mass and function, especially in older adults (see Table 2 [330]). Importantly, combining aerobic exercise with resistance training is more effective in reducing insulin issues in the obese population with metabolic syndrome and improving health in many other disorders [330,331,332]. Exercise substantially and quickly alters the physiological background since ~9800 molecular analytes (proteins, transcripts, metabolites, and lipids) changed expression levels from a single round of aerobic exercise [333]. A similar comparative metabolomics study reported that 666 and 708 metabolites were altered in blood plasma samples following aerobic and resistance exercises, respectively [334].

Since skeletal muscle is the largest tissue in the human body, it releases many myokines into the peripheral circulation following exercise [335,336]. Numerous studies have compared the effects of aerobic exercise and resistance training on myokine/exerkine production. A partial list of myokines detected in blood following an episode of exercise is provided in Table 3. Interestingly, some myokines respond preferentially to a specific type of exercise, while others respond similarly regardless of the exercise type. Many of the exerkines included in Table 3 were described as putative neuroprotective exerkines, suggesting that both forms of exercise can promote exerkine release, promoting a neuroprotective effect.

García-Hermoso et al. [371] conducted a comprehensive review of the effect of exercise on type 2 diabetes, involving more than 2000 patients and control groups. The exercise routines in these studies encompassed a wide range, including aerobic exercise, high-intensity interval training, resistance training, and exercise with two modalities. The duration of these routines varied from 8 weeks to 96 weeks, with training sessions held from one to five times per week [371]. The meta-analysis from this study showed that any form of regular exercise alters the blood levels of many exerkines, including adiponectin, fetuin-A, FGF-21, IL-6, IL-10, leptin, resistin, and TNF-α, in patients with type 2 diabetes compared to control groups. Specifically, aerobic exercise, resistance training, or high-intensity interval protocols, performed at moderate-to-vigorous intensity for more than 24 weeks with three sessions per week and lasting more than 60 min per session, yielded the most promising results for type 2 diabetes patients [371].

The impact of resistance training on neuroprotective factors has yet to be studied as thoroughly as aerobic exercise. Rodríguez-Gutiérrez et al. [372] performed a systematic review and meta-analysis of resistance training and changes in levels of IGF-1, BDNF, and VEGF. Thirty randomized clinical trials were included in their study [372]. They found a significant effect of resistance training on increased levels of IGF-1 but not for BDNF (VEGF was not determined due to the lack of publications). Their study supports a neuroprotective effect of resistance training in middle to late adulthood primarily due to the increased release of IGF-1 [372].

### 4.2. Age

Advanced age is the most significant risk factor for developing PD [373]. Mild cognitive impairment (MCI) is linked to an irregular aging pattern that can lead to dementia, with a risk of developing further into Alzheimer’s disease (AD) [374]. Cognitive changes can also occur in PD; thus, finding treatment for MCI is essential for both AD and PD. To better understand the mechanism of exercise in treating MCI, Tsai et al. [375] compared aerobic exercise and resistance training in neurocognitive performance in older adults with MCI. They also measured changes in several exerkines in both forms of exercise. Aerobic exercise significantly increased BDNF and IGF-1 and a minor increase in VEGF, while resistance training only increased IGF-1 [375]. Both aerobic exercise and resistance training improved neurocognitive performance. They concluded that neuroprotective exerkines were operational in older adults with MCI, which suggests that exercise-dependent plasticity is possible [375].

In a unique approach, Kettinen et al. [376] investigated cognitive decline in aging populations by employing age-appropriate cognitive demanding aerobic exercises. Healthy older golfers performed these exercises, and the study was particularly interested in cognitive changes in three groups: 18 holes of golf, 6 km of Nordic walking, or 6 km of regular walking [376]. The researchers also tested for changes in BDNF and CTSB. Interestingly, despite not detecting any changes in the exerkines measured, all three exercise groups showed improved cognitive functions. Furthermore, the study revealed that Nordic and regular walking enhanced executive functions. This research addresses a crucial question: can we design age-appropriate exercises to enable an aging population to improve their cognitive function [376]? Moreover, this study further underscores the importance of exercise, suggesting that it may be more beneficial than doing nothing.

### 4.3. Muscle Wasting

Frequently, with advanced aging, adults can suffer a significant loss of skeletal muscle mass, which results in a disorder called sarcopenia [377,378]. Sarcopenia is found to be increased in PD when compared to other aged-match non-PD patients. Since many exerkines are generated in skeletal muscle, Kwon et al. [379] reported that the following myokines, regulated by exercise, were altered by aging: apelin, BAIBA, BMP-7, decorin, IGF-1, IL-15, irisin, SDF-1, sestrin, SPARC, and VEGF were decreased, while IL-6 and myostatin were increased. Aerobic exercise increased apelin, BAIBA, IL-15, IL-6, irisin, SDF-1, sestrin, SPARC, and VEGF expression, while resistance training enhanced BMP-7, decorin, IGF-1, IL-15, IL-6, irisin, and VEGF expression [379]. Both forms of exercise downregulated myostatin. They concluded that it is critical to develop combined aerobic exercise and resistance training programs for aging adults [379]. Likewise, Piccirillo [380] and Graham et al. [381] provide further mechanistic insight into the role of exercise and myokines in preventing muscle wasting.

### 4.4. Sedentary Lifestyle

PD can promote a sedentary lifestyle in older adults [382]. To address the sedentary lifestyle (this study did not pertain to PD), MacNeil et al. [383] studied 68 healthy younger and older volunteers and measured 44 low-abundance growth factors, cytokines, and chemokines from their blood. They found that long-term physically active people had a broader induction of factors post-exercise, irrespective of age [383]. They concluded that sedentary older adults have a lower aerobic capacity and muscle function, which implies that the lack of change in myokines released into the bloodstream was likely due to the reduced force of muscle contractions [383]. This study reinforces the notion that well-planned exercise programs can potentially help manage a sedentary lifestyle.

Sustained moderate-to-vigorous exercise leads to enhanced plasticity in the motor cortex [384]. McDonnell et al. [385] wanted to know the effect of a single exercise session. They found that a single session of light exercise promoted a neuroplastic response, which could enhance the effectiveness of motor learning [385]. Furthermore, Steib et al. [386] demonstrated that a single session of moderately intense cycling led to rapid improvement in motor skill consolidation in patients with PD. These studies suggest that introducing exercise to a PD patient who has been sedentary can lead to rapid improvements in motor skills and learning deficits, providing a strong sense of encouragement and motivation.

In the next section, we start with a description of the neuroprotective theory. From there, we provide a road map of exerkines and how they potentially enable the brain to protect itself. In the case of PD, exercise and exerkines may unite to join an offensive attack, promoting regeneration (or neuroplasticity) in the brain.

## 5. The Neuroprotective Theory to Slow the Progression of Parkinson’s Disease

“Your age is the sum total of your physical condition, the condition of your mind, and how you feel.” Jack LaLanne (1914–2011; LaLanne hosted the first and longest-running syndicated fitness television program in the U.S., from 1951 to 1985)

### 5.1. Assessing the Neuroprotective Theory

Neuroprotection is the strategy for safeguarding the CNS from neuronal cell damage caused by acute injury (e.g., stroke or trauma) or chronic neurodegenerative diseases [387]. Therefore, the neuroprotective theory includes the process and components derived from exercise, which creates the physiological setting for the formation and release of exerkines into the blood circulatory system [388,389]. Following this is the subsequent crosstalk between the brain and multiple organs/tissues, instructing the various exerkines to migrate to their target cells in the CNS [50,390], thus beginning the ligand (exerkines)/receptor-binding site interaction, which leads to the activation of numerous cell-surface receptors, changing the properties of the neurons, and then producing/secreting their own bioactive molecules. The neuroprotective theory can promote either neuroprotection or neuro-restoration of damaged/dead neurons [391].

Increasing evidence indicates both preventive and therapeutic functions for exerkines [392,393]. What is intriguing about the neuroprotective theory is that most exerkines do not originate in the brain; by contrast, skeletal muscle, liver, and white adipose tissue likely account for the influx of exerkines to the brain [394]. The neuroprotective theory functions well for many individuals who will never develop PD or another neurodegenerative disorder. However, for some, factors include exposure to neurotoxins, alterations in immunological or inflammatory systems, genetic misprinting, mitochondria dysfunction, or numerous other possibilities that shift the equilibrium to favor disease over health [387]. Although a neurodegenerative disorder presents a life-or-death scenario, the exercise/exerkines response is less robust than the full burst impact of the polymorphonuclear leukocytes in acute inflammation attacking/phagocytosing an extracellular microbial invader [395], the mononuclear leukocytes of chronic inflammation again matched up against a foreign invading substance [396] or the ultimate firepower generated by a T-cell/B-cell-directed immune attack of an invading organism [397]. Perhaps the protective shield of neuroprotection is more of a gentle salve soothing a vital tissue/organ, not the sheer extreme firepower found in our immunological/inflammatory machinery. However, the equilibrium shifts when one chooses a more intense and sustained exercise program, creating an *in vivo* scenario of increased neuroprotection and, over time, the potential to repair and regenerate neuronal tissue by neuroplasticity.

The neuroprotective theory involves exerkines with many different biological roles. However, for this physiologic process to succeed, it is essential to appreciate what it is up against when encountering the development and progression of PD [30,398]. As shown in Figure 1, several environmental and genetic missteps contribute to the genesis of PD. Furthermore, it is likely a combination of missteps in these molecular processes that provide for the development of PD, therefore creating a complicated target for the neuroprotective machinery to manage. One assumes that the neuroprotective factors provided by exercise and exerkines participate in maintaining neuronal health, circuitry, architecture, and normal function.

### 5.2. Initiation Pathways for Aerobic Exercise and Resistance Training

Aerobic exercise aims to enhance skeletal muscle capacity for aerobic metabolism and to resist fatigue [330,399,400]. This is achieved through an increase in the number of mitochondria and types I and II muscle fibers in skeletal muscle [401]. The process of aerobic exercise triggers PGC1-α synthesis [402], which in turn promotes the expression of irisin (derived from FNDC5) in skeletal muscle [229]. As described by Wrann et al. [231], aerobic exercise could have the same effect on the brain (hippocampus), specifically through the PGC-1α/FNDC5/BDNF pathway. This novel finding suggests that aerobic exercise could enhance the transcription of *Pgc1a* and *Erra*, activating gene expression of *Fndc5* [231]. From there, FNDC5 (irisin) acts as a positive regulator of BDNF expression, potentially offering a new avenue for neuroprotection in PD.

In contrast to aerobic exercise, resistance training stimulates skeletal muscle growth by promoting myofibrillar protein synthesis [330,403,404,405]. This process leads to muscle hypertrophy, the increase in myofibrillar volume in type II skeletal muscle fibers [406,407,408]. To achieve hypertrophy, skeletal muscle overexpresses IGF-1 and activates the PI3/Akt signaling pathway [336,380]. Similarly, in the brain, IGF-1 binds to its receptor, IGF-1R, along with other growth factors like BDNF (which binds to TRkB) and VEGF (which binds to VEGFR) [409,410]. Together, they trigger the PI3/Akt pathway [410]. Downstream of PI3/Akt pathway are several targets in the brain, and mTOR activation promotes neurogenesis [410].

Combining aerobic exercise with resistance training offers neuroprotection for the brain [27,73,150,411,412,413,414] and benefits the entire body and other tissues/organs [415,416,417]. This powerful synergy of both types of exercise underscores the potential for comprehensive health improvement, whether in the absence or presence of PD.

### 5.3. Neuroprotective Action from Myokines in Model Systems of Parkinson’s Disease

Exercise and exerkines have been well studied in some neurodegenerative diseases (e.g., AD) and neurocognitive disorders (e.g., MCI), but not as much in PD. The following *in vitro* system was used to study the neuroprotective role of myokines in model systems of PD: cultured neuronal cells (SH-ST5Y cells or PC12 cells), adding a neurotoxin to initiate a PD-like scenario (6-OHDA, MPTP, or MPP+), and then adding myokines (apelin, FGF21, or IGF-1) exogenously (Table 4). For each myokine tested, the studies found that myokines reduced cell death (apelin, FGF21, and IGF-1), increased α-synuclein clearance (apelin, FGF21), and promoted an anti-inflammatory response (apelin, FGF21, IGF-1). Furthermore, similar results were obtained when using mice and rats, though not directly involving exercise, which supports the hypothesis that myokines are neuroprotective in PD.

### 5.4. Neuroprotective Components Provided by Exerkines

**Figure 4:**
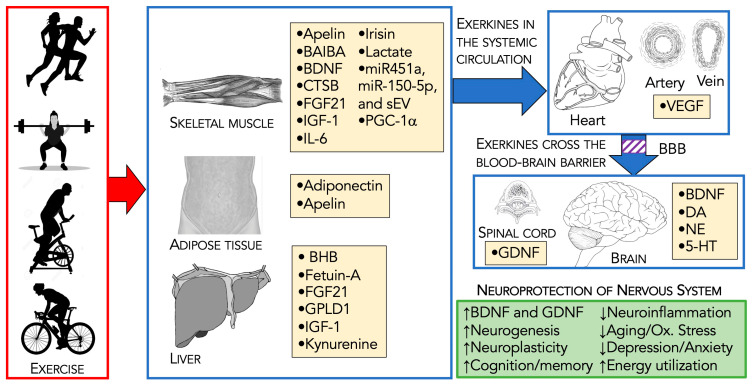
Potential role of neuroprotective exerkines to slow the progression of Parkinson’s disease.

What the brain senses due to exercise still needs to be fully understood. A part of it is an endocrine communication system (“crosstalk”) between the brain and other organs with exerkines, and another aspect must be the physiological changes in the body because of exercise (e.g., oxidative changes or the changes induced early on by exerkines interacting with their target receptors). Figure 4 provides a composite view of the exercise-induced exerkines described in Section 3 and the collective neuroprotective process in PD. Skeletal muscle provides the most significant number of exerkines to the neuroprotective process, followed by the liver and white adipose tissue [426]. The role of exerkines in the neuroprotective theory provides several powerful physiological reactions to manage the numerous PD symptoms. We describe some of the potential intervention points exerkines supply during this process.

Anti-Aging and Reduction in Oxidative Stress—Irisin is a protein that activates another protein called Nrf-2 in liver, heart, and endothelial cells [427]. Nrf-2 helps regulate nitric oxide signaling in blood vessel cells [428], which reduces the amount of reactive oxygen species in the body. This helps to increase the amount of superoxide dismutase in the brain, which protects against oxidative damage. In older mice, GPLD1 has been found to promote the growth of new brain cells and the expression of BDNF, which is essential for learning and memory [221]. Additionally, BAIBA activates two different signaling pathways in the body, including the AMPK and PI3K/Akt pathways. This activation helps to protect cells against oxidative stress [202,255].

Depression and Anxiety—In a study conducted on mice with symptoms like chronic depression, aerobic exercise increased the production of PGC-1α, which, in turn, up-regulated AMPK (a critical component of energy balance) [429]. This led to significant improvement in depression-like behavior. Additionally, kynurenine aminotransferase was also upregulated, and the ratio shifted in favor of kynurenic acid over Kyn, resulting in a reduction in depression [306].

Energy Utilization in Mitochondria—Mitochondria play a crucial role in producing ATP, which supports the maintenance of neuronal cells and facilitates neurotransmitter communication [430]. Heo et al. [431], on reviewing exerkines and brain mitochondria, suggest that BDNF, IL-6, BHB, lactate, adiponectin, irisin, and FGF21 all impact mitochondrial bioenergetics and energy production.

Neurogenesis—Generating neurons in the adult brain requires activating quiescent neuronal stem cells, which BDNF can achieve in the hippocampus [432,433]. IGF-1 can promote neurogenesis and help coordinate the impact of BDNF and VEGF in the brain [247,434].

Neuroinflammation—Neuroinflammation refers to the host defense system components that play a crucial role in Parkinson’s disease (PD) pathogenesis [435]. Adiponectin helps to control microglia function by promoting anti-inflammatory responses through PPAR-γ signaling [436,437]. Apelin-13, a derivative of apelin, has been found to inhibit microglia and astrocyte activation, reduce IL-1β and TNF-α expression, and cause hippocampal BDNF/TrkB expression deficit in rats with Alzheimer’s disease [438]. BHB effectively blocks inflammasome activation in PD [439]. IL-6 is a potent anti-neuroinflammatory agent as it upregulates key anti-inflammatory cytokines such as IL-10 and IL-1 receptor antagonists [227].

Neuroplasticity—Adult neuroplasticity is described as changes in the strength of excitatory and inhibitory synapses, while various attempts to regenerate connections have met with limited success [440]. In a comprehensive review of neuroprotective exerkines, Vints et al. [441] provided evidence for long-term synaptic potentiation, a component of the signaling pathways that promote neuroplasticity for BDNF, IGF-1, irisin, CTSB, apelin, adiponectin, kyn, lactate, and BHB.

Tissue Regeneration—Exercise and exercise-induced exerkines aim to promote a scenario that can protect, repair, or regenerate the brain of someone with PD. Exercise exerts a physiological response [47] while exerkines support, in part, biochemical and molecular signaling responses (two examples are given) [48]. One such example is the activation of the PI3K/Akt signaling pathway [410], a crucial factor in response to exerkines, notably IGF-1 [224], VEGF [247,248,249], and BDNF [265,266], which promotes neuronal survival, proliferation, and maturation. The expression of PGC-1α leads to activation of the AMPK/SIRT1/PGC-1α signaling pathway [410]. The role of PGC-1α is essential to mitochondrial biosynthesis and is also involved in cell proliferation and differentiation, which are critical processes in exercise-induced neural regeneration [442]. PGC-1α also forms heterodimer complexes with PPARs and estrogen-related receptors, thus widening the impact of PGC-1α exercise-induced neural regeneration [443].

Gut Microbiome–Brain Interaction—There is evidence for a gut microbiome–brain conduit in the pathogenesis of PD [444,445,446,447]. The peripheral nervous system, notably the vagus nerve, is the communication link between the gut and brain. Alterations in the gut microbiome produce toxins that help form α-synuclein aggregates. Then, presumably by a prion-like process, the α-synuclein aggregates are transferred into the peripheral nervous system. Thus, pathological changes to the gut microbiome may contribute to PD [444,445,446,447]. Likewise, exercise can positively influence the microbiome, promoting an anti-inflammatory response [448,449,450]. A part of the neuroprotective theory may be the positive impact of exercise in generating an anti-inflammatory gut microbiome that signals through the peripheral and central nervous systems to augment the neuroprotective exerkines in slowing the progression of PD.

Summary—Combined with exercise, the following exerkines (listed alphabetically) demonstrate potential for protecting the brain against the pathological missteps caused by PD: apelin, BHB, BDNF, FGF21, GDNF, IGF-1, IL-6, irisin (FNDC5), lactate, PGC-1α, and VEGF.

## 6. Strengths, Limitations, and Challenges

“We must turn to nature itself, to the observations of the body in health and in disease to learn the truth.” Hippocrates (460–375 BCE) [2]

Strengths—One of the goals of this review was to use previous work on PD as a foundation to help guide the narrative topics. From these studies/reviews, we determined that there were some critical unanswered questions about PD, therapy, exercise, and exerkines. Can exercise target the complex pathophysiology of PD [451,452]? Alternatively, is exercise promoting a healthier total self physiologically to allow for a more robust reaction against PD [453]? Exercise is now considered a primary component in treating PD; however, a better appreciation of specific molecular and cellular pathways is needed [53,454]. Can exercise further improve cognition or executive function deficits [455]? Is aerobic exercise or resistance training better for treating the symptoms of PD [45,154,411,456,457,458,459,460,461]? Can exercise be personalized by healthcare providers to match the variable presentation of PD from person to person [451,462,463]? Another goal of this review was to unite the complex field of assessing exercise for treating PD with the critical role of exercise-induced exerkines to promote the benefit of exercise.

Limitations—This paper was written as a narrative review, not as a systematic review with a meta-analysis study. Thus, some studies cited here are only sometimes consistent about a specific type of exercise linked to improved motor function or changing levels of exerkines. These differences are likely due to testing design, sample detection limits, and whether the study was large enough to gain statistical significance. In many cases, the exerkine studies we described were based on cardiovascular diseases or neurocognitive disorders, not PD. While much evidence supports the role of exercise and exerkines in providing neurological-based protection, there are only a few papers on exerkines in PD (most often without exercise) [252,268,269,275,287,293,301,303,310,418,419,420,421,422,423,424,425]. Interestingly, a clinical trial is underway with GDNF therapy in PD [464].

Challenges—As asked by Wrann et al. [231], how does the brain sense exercise? The answer is still pending. The difficulty in answering this all-important question is likely because exercise changes the body. Identifying the signals that lead to the cross-talk between multiple tissues will help provide a molecular exercise map [465]. Exercise studies today use many different types of exercise, and some include aerobic exercise [466], resistance training [460], exercise alone and in groups [414], and even high-intensity interval training [93]. Many studies have a variable time of study with different intensities. These variabilities further complicate the best-targeted exercise for treating a disorder [467]. PD is a complex disorder, started by a multitude of factors, with numerous symptoms and a variable degree of progression, which means that patients enrolling in exercise studies may require different levels of exercise training depending upon their disorder, and their baseline fitness may be different. However, performing these kinds of studies will provide us with knowledge and a spectrum of information about how to use exercise to manage the complexity of PD.

## 7. Conclusions

“The use of exercise has had an important share in the treatment of disease since Hippocrates used it at the sanitarium at Cos, and Galen advocated it in words that are as true now as they were eighteen hundred years ago…” R. Tait McKenzie, M.D. (1909) in “Exercise in Education and Medicine” [468].

The concept of exercise as medicine is ancient [1,2,468]. We are currently unraveling the molecular processes and pathways to how exercise provides a protective shield to our tissues/organs. In this narrative review, we described the importance of exercise and various exercise programs to best support each PwP. We presented the ability of exercise-induced exerkines to provide neuroprotection, reducing the risk of PD and offering neuroprotection and possibly neuro-restoration for those afflicted with PD.

The diverse nature of PD and the varying progression rates found in PwP necessitate tailored management strategies. Additionally, PwP may present with different levels of physical fitness, further complicating their physical and mental health management. We wrote this review to assist Care Teams (both professional and personal) in understanding the role and application of exercise (aerobic exercise, resistance training, neuromotor exercise programs, and stretching/flexibility exercises) and in developing comprehensive and personalized management plans for PwP. Furthermore, by highlighting the molecular and physiological properties of exerkines, the Care Teams and PwP can gain knowledge and appreciate this neuroprotective process empowered by exercise. Finally, as our understanding of the role of exerkines expands and evolves, this will provide scientific, physiological, and pharmacological platforms to improve the health of PwP further.

## Figures and Tables

**Table 1:** Representative exercise programs for people with Parkinson’s disease.

Individual Profile	Aerobic Exercise	Resistance Training	Neuromotor Rehabilitation Programs	Flexibility/Stretching Exercises
Eighty-year-old male with advanced PD, ambulates with a rollator and requires caregiver support. He experiences some “off times” in which his medication becomes less effective. He is highly motivated with a great family and friend network.	Rides a NuStep or recumbent bike for 40 min 3×/week. Target HR of 60–70% HRmax	Participated in a seated strengthening program with use of resistance bands for upper body and supervision of a caregiver 2×/week. Completed during “on time” of medication cycle.	Currently receiving PT once every two weeks for maintenance PT. Also receives restorative PT periodically throughout the year. Goal of working on weight shifts with his rollator alongside a trained family member 3×/week.	Participates in daily stretches in his bed before getting up in the morning. Goal of taking a Dance for PD class online 1×/week.
Retired 70-year-old female with short term memory loss who enjoys staying active so she can spend time with her children and grandchildren	Walks 1–2 miles every day (when it is not raining). Target RPE for walks is 6/10.	Goes to the gym 2×/week and uses resistance machines after participation in exercise classes.	Receives restorative PT approximately 1–2×/year. Goal of taking 1–2 classes at her gym each week. (Varies between PWR!Moves, tai chi and Dance for PD) Sometimes her daughter will join her.	Participates in 10 min of home stretches as instructed by PT 5×/week.
Sixty-five-year-old female who was diagnosed one year ago. She has lived a lifetime of being active and is very willing to try to explore exercise options. She is still working and will retire in 1–2 years.	Walks on a treadmill 3×-week with goal of 75% max HR and plays tennis 2×/month	Works out in her home gym using free weights 2×/week	Participates in benchmark assessments 2×/year and restorative PT as needed Participates in a mix of the following activities 1–2×/week: tennis, tai chi, golf, PWR!Moves classes	Incorporates 15 min of stretching as a cool down after her resistance workouts, 2×/week
Recently diagnosed retired 76-year-old male with high blood pressure (controlled with medication) who is relatively sedentary, resistant to begin exercising but willing to give it a chance. He checks his BP daily.	Walks his family dog 3×/week for 20 min. He is working towards a goal of walking for 25 min.	Goal of participating in Rocksteady boxing 1×/week, Goal of completing body weight resistance exercises 2×/week during the commercial breaks of a television show he watches every day	Referred to PT to help establish an exercise program and address balance. PT will also instruct on modifications as needed to improve safety with exercise in individuals with high blood pressure.	Goal of incorporating 10 min of stretches after he gets up in the morning Goal of completing PWR!Moves seated mobility exercises 3×/week during the commercial breaks of a television show he watches every day
Retired 70-year-old male originally diagnosed at age 59 who experiences right side stiffness, right arm tremor, controlled with Carbidopa/Levodopa, NeuPro patch; follows an integrated approach to treatment.	Rides his Peloton bike for 45 min with goal of 80% HRmax 3×/week; 3–5 mile power walk 1×/week	30–45 min weightlifting on major muscle groups 2×/week	LSVT BIG, and regularly works with a PT for any sports-related injury	Daily stretching, and 2× week aim for PWR!Moves or RockSteady Boxing. Participates in golf 2–3×/week.
Forty-one-year-old female who was diagnosed at age 39. She has two children ages 10 and 12 and is still working. She was previously active but not a formal exerciser. Over the past two years has worked with her healthcare team to start an exercise program that emphasizes aerobic components.	She initially used the couch to 5K program to begin running. Now participates in a running group and runs 3–5 miles 2–3×/week with target HRmax of 80%, also walks 20–30 min 2–3×/week. (She will often walk during her children’s soccer practice.)	Goes rock climbing with her son 2×/month. Goal of participating in 30 min of strength training 2×/week. She uses an online fitness membership to guide her workouts.	She worked with a PT to initiate an exercise program and participates in benchmark testing 2×/year. Returns to PT as needed to adjust exercises. Goal of participating in yoga 2×/week in addition to running.	Goal of completing a 15 min yoga video 2×/week. Goes rock climbing with her son 2×/month.

**Table 2:** Overview of some exerkines with neuroprotective properties.

Name	Source Tissue(s)	Target Tissue(s)	Example(s) of Biological Action	Reference(s)
Adiponectin	WAT	Brain, heart, liver, muscle	Anti-inflammatory, glucose and fatty acid metabolism	[195,196,197,198]
Apelin	Skeletal muscle, WAT	Brain, bone, skeletal muscle	Anti-apoptotic	[48,199]
BAIBA	Skeletal muscle	Many sites	Metabolic regulator, anti-inflammatory, anti-oxidative	[200,201,202]
BHB	Liver	Many sites	Improves fuel utilization, anti-inflammatory	[203,204,205]
BDNF	Brain, skeletal muscle	Brain	Enhances synaptic plasticity, and cell growth	[206,207,208,209]
CTSB	Skeletal muscle	Brain	Neurogenesis, memory	[210,211]
Fetuin-A	Liver	Liver, brain	Neuroprotective, anti-inflammatory	[212,213,214]
FGF21	Many tissues, liver, WAT	Many tissues, heart, bone, gut, brain, WAT	Improves fuel utilization (glucose, lipid)	[215,216,217]
GDNF	Spinal cord	Brain	Maintenance of spinal motor neurons and midbrain dopaminergic neurons	[218,219,220]
GPLD1	Liver	Brain	Improves neurogenesis and cognition	[221,222]
IGF-1	Liver, skeletal muscle, and other tissues	Brain, many sites	A mediator of brain health following exercise including neurogenesis and cognitive improvement	[223,224,225]
IL-6	Skeletal muscle	Many sites	Energy sensor, anti-inflammatory in brain	[226,227,228]
Irisin (FNDC5)	Skeletal muscle	WAP, bone, β-cells, brain	Promotes neuronal health	[229,230,231]
Lactate	Skeletal muscle	Many tissues, including CNS	Brain fuel source	[232,233,234]
Lac-Phe	Epithelial cells in gut and kidney	Many tissues, including CNS	Suppresses feeding and obesity	[235,236,237]
mIR451a/mIR-150-5p/sEV	Skeletal muscle and other tissues	Many tissues including CNS	Protects against depression (miR-451a) and anxiety (miR-150-5p)	[238,239,240]
Monoamine neurotrans-mitter (DA, NE, 5-HT)	Brain	Brain	DA—Motor control, learning, executive function; NE—stress resistance memory, cognition; 5-HT—relieve anxiety, stress protection, cognition	[241,242,243]
PGC-1α/ Kynurenine	Skeletal muscle, liver, and other tissues	Skeletal muscle, and brain	PGC-1α activates kynurenine aminotransferase, switching the ratio of kynurenine (neurotoxic) to kynurenic acid (neuroprotective)	[244,245,246]
VEGF	Skeletal muscle and other tissues	Brain endothelium and other tissues	Promotes angiogenesis and exercise-induced neurogenesis	[247,248,249]

**Table 3:** Exercise-induced myokines detected in blood following aerobic exercise or resistance training. Arrows describe an increase (↑) or decrease (↓) in exerkine levels from exercise.

Aerobic Exercise [Reference(s)]	Resistance Training [Reference(s)]
Apelin ↑ [253,337] BAIBA ↑ [338] BDNF ↑ [339,340] CTSB ↑ [341] FGF21 ↑ [342] Fractalkine ↑ [343] IGF-1 ↑ [285,344] IL-6 ↑ [345,346] IL-15 ↑ [347] Irisin ↑ [229] Lactate ↑ [348] LIF ↑ [349] MCP-1 ↑ [343] Musclin ↑ [350] Myonectin ↑ [351] Myostatin ↓ [352] PGC-1α ↑ [353,354] SDF1 ↑ [355] SPARC ↑ [356] RANTES ↑ [216] VEGF ↑ [357]	Angiopoietin-like 4 ↑ [358] BDNF ↑ [359] BMP7 ↑ [360] CTSB ↑ [361] Decorin ↑ [362] FGF21 ↑ [363,364] IGF-1 ↑ [283,365] IL-6 ↑ [346] IL-15 ↑ [366] Irisin ↑ [364,367] Lactate ↑ [368] Myostatin ↓ [352] PGC-1α ↑ [369] VEGF ↑ [370]

**Table 4:** Neuroprotective properties of myokines using neuronal cells and animal models of PD *.

Myokine	Experimental Model	Mechanism of Action	Physiological Function	Reference
Apelin-13	SH-ST5Y cells + MPP^+^ + apelin-13	↑ ERK1/2 phosphorylation ↓ ER stress	↓ Cell death	[418]
Apelin-36	SH-ST5Y cells + MPP^+^ + apelin-36	↑ PI3K/Akt/mTOR autophagy	↑ α-Synuclein clearance	[419]
Apelin-36	Mice + MPTP + apelin-36	↑ GSH and SOD ↓ Caspase-3	↓ Anti-oxidative stress	[420]
Apelin-13	SH-ST5Y cells + 6-OHDA + apelin-13	↑ PI3K ↓ Caspase 3 ↓ Cytochrome c	↓ Cell death	[421]
FGF21	Mice + MPTP + FGF21 SH-ST5Y cells + MPTP + FGF21	↑ SIRT1-autophagy	↑ α-Synuclein clearance	[422]
FGF21	Mice injected with MPTP + FGF21 SH-ST5Y cells + MPTP + FGF21	↑ BCL2/Bax ↓ Caspase 3 cleavage and JNK phosphorylation	↓ Cell death	[423]
FGF21	Primary dopaminergic neurons + MPP^+^ + FGF21	↓ IL-1, IL-12, IFN-γ, and TNF-α ↓ Astrocyte and microglia activation	↑ Anti-inflammation	[423]
IGF-1	PC12 cells + 6-OHDA + IGF-1	↓ Caspase-3 activation, and PARP cleavage	↓ Cell death	[424]
IGF-1	Rats + 6-OHDA + IGF-1	↑ PI3K/Akt	↓ Cell death	[425]
IGF-1	Rats + 6-OHDA + IGF-1	↑ ERK 1/2/CREB ↑ Akt/GSK3α/β/ β-catenin	↓ Cell death	[287]
IGF-1	PC12 cells + 6-OHDA + IGF-1	↑ HO-1	↓ Anti-oxidative stress	[424]

* The table is partly based on Lee et al. [19]. Apelin-13 and -36 are modified forms of apelin. Arrows describe an increase (↑) or decrease (↓) in the biological activity measured from the experiment.

## Abbreviations

AD	Alzheimer’s disease
ALS	amyotrophic lateral sclerosis
AMPK	AMP-activated protein kinase
BAIBA	beta-aminoisobutyric acid
BBB	blood–brain barrier
BDNF	brain-derived neurotrophic factor
BHB	beta-hydroxybutyrate
Bpm	beats per minute
CNS	central nervous system
CTSB	cathepsin B
DA	dopamine
ERK 1/2	extracellular signal-regulated kinase 1/2
FGF21	fibroblast growth factor 21
FNDC5	fibronectin type III domain-containing Protein 5
GDNF	glial cell line-derived neurotropic factor
GPLD1	glycosylphosphatidylinositol-specific phospholipase D1
GPCR	G protein-coupled receptor
% HR_max_	% maximal heart rate
HCAR2	hydroxycarboxylic acid receptor 2
H&Y	Hoehn and Yahr scale of PD stage
6-OHDA	6-hydroxydopamine
IGF-1	insulin-like growth factor 1
IL-1, -6, -10	interleukin-1, -6, -10
Kyn	Kynurenine
Lac-Phe	Lactoylphenylalanine
miR	microRNA
MPTP, MPP^+^	1-methyl-4-phenylpyridinium (Inactive/active forms)
MCI	mild cognitive impairment
p38 MAPK	p38 mitogen-activated protein kinase
ND	neurodegenerative
NE	norepinephrine
NF-κB	nuclear factor kappa-light-chain-enhancer of activated B cells
OT	occupational therapist
PD	Parkinson’s disease
PwP	people (person)-with-Parkinson’s
PGC-1α	peroxisome proliferator-activated receptor gamma coactivator 1-alpha
PPARα/δ/γ	peroxisome proliferator-activated receptor alpha/delta/gamma
PI3K/AKT/mTOR	phosphatidylinositol 3-kinase/protein kinase B/mammalian target of rapamycin
PT	physical therapist
PTSD	Post traumatic stress disorder
Irisin	proteolyzed fragment of FNDC5
PAI-1	plasminogen activator inhibitor-1
5-HT	serotonin
SIRT1	sirtuin 1
SLP	speech–language pathologist
sEV	small extracellular vesicles
TBI	Traumatic brain injury
TrkB	tropomyosin receptor kinase B
TNF-α	Tumor necrosis factor-alpha
UPDRS	Unified Parkinson’s Disease Rating Scale
VEGF(R)	vascular endothelial growth factor (receptor)
WAT	white-adipose tissue
